# Cullin3 - BTB Interface: A Novel Target for Stapled Peptides

**DOI:** 10.1371/journal.pone.0121149

**Published:** 2015-04-07

**Authors:** Ivan de Paola, Luciano Pirone, Maddalena Palmieri, Nicole Balasco, Luciana Esposito, Luigi Russo, Daniela Mazzà, Lucia Di Marcotullio, Sonia Di Gaetano, Gaetano Malgieri, Luigi Vitagliano, Emilia Pedone, Laura Zaccaro

**Affiliations:** 1 Institute of Biostructures and Bioimaging, C.N.R., Napoli, Italy; 2 Institute of Crystallography, C.N.R., Bari, Italy; 3 Second University of Napoli, Caserta, Italy; 4 Interuniversity Centre for Research on Bioactive Peptides (CIRPEB), Napoli, Italy; 5 Department of Molecular Medicine, La Sapienza University, Roma, Italy; Molecular Biology Institute of Barcelona, CSIC, SPAIN

## Abstract

Cullin3 (Cul3), a key factor of protein ubiquitination, is able to interact with dozens of different proteins containing a BTB (Bric-a-brac, Tramtrack and Broad Complex) domain. We here targeted the Cul3–BTB interface by using the intriguing approach of stabilizing the α-helical conformation of Cul3-based peptides through the “stapling” with a hydrocarbon cross-linker. In particular, by combining theoretical and experimental techniques, we designed and characterized stapled Cul3-based peptides embedding the helix 2 of the protein (residues 49–68). Intriguingly, CD and NMR experiments demonstrate that these stapled peptides were able to adopt the helical structure that the fragment assumes in the parent protein. We also show that some of these peptides were able to bind to the BTB of the tetrameric KCTD11, a substrate adaptor involved in HDAC1 degradation, with high affinity (~ 300–600 nM). Cul3-derived staple peptides are also able to bind the BTB of the pentameric KCTD5. Interestingly, the affinity of these peptides is of the same order of magnitude of that reported for the interaction of full-length Cul3 with some BTB containing proteins. Moreover, present data indicate that stapling endows these peptides with an increased serum stability. Altogether, these findings indicate that the designed stapled peptides can efficiently mimic protein-protein interactions and are potentially able to modulate fundamental biological processes involving Cul3.

## Introduction

Protein-Protein interactions play fundamental roles in living organisms. The ability to modulate these interactions paves the way for the regulation of key physiopathological processes. Different structural elements typically contribute to protein-protein interface. Since these interfaces frequently involve α-helices, peptides mimicking interacting helices represent a valuable tool for modulating protein-protein contacts. However, α-helical regions eradicated from their protein context tend to lose their structure, thus limiting the efficacy of the peptide mimetic approaches.

In recent years, different strategies have been developed to stabilize α-helical conformations. By using ring closing metathesis (RCM) developed by Blackwell and Grubbs [1], the Verdine group has optimized α-helix stabilization known as *stapling* introducing synthetic alpha, alpha-di-substituted non-natural amino acids of different lengths and stereochemistries [2]. Although the effectiveness of this stapling approach has been occasionally questioned [3], it has been shown that it can greatly improve the pharmacologic performance of peptides, enhancing their target affinity, resistance toward proteolytic degradation, and serum-half-life, and, most significantly, their cellular uptake [2] thereby increasing the bioavailability and their potential as therapeutic agents. This technology has been already used successfully in different biological contexts [4] such as p53 pathways [5], NOTCH pathways [6], BCL pathways [7], estrogen activation [8], cholesterol efflux [9] and in targeting HIV [10].

In this work the stapling approach has been extended to the interaction of Cullin 3 (Cul3) with proteins containing BTB/POZ (Bric-a-brac, Tramtrack and Broad Complex/Pox virus and Zinc finger) domains. Cul3 is an essential component of the CRL3 system (Cullin 3-Ring ubiquitin Ligases) which facilitates the transfer of ubiquitin to protein substrates [11, 12]. CRL3 is an important regulator of cellular and developmental processes. Its alteration is linked to several human diseases [13]. In performing its function, Cul3 simultaneously binds the Ring protein that carries the E2 enzyme and the substrate adaptor. It has been shown that the Cul3 adaptor partners share a common structural BTB domain [14]. It is important to note that Cul3 is able to interact with dozens of BTB-containing proteins, which potentially bind a variety of different substrates to be ubiquitinated. Therefore, for a large number of proteins, the molecular recognition between Cul3 and BTB domains is a crucial step for protein ubiquitination and regulation. In this scenario, the development of molecules that are able to interfere with Cul3/BTB interaction could represent an important step for regulating a variety of different processes. Here we targeted the interaction of Cul3 with the BTB containing protein KCTD11, one of the best characterized members of the emerging class of multidomain proteins denoted as KCTD (Potassium Channel Tetramerization Domain containing proteins) [15–17]. These proteins present a N-terminal BTB domains which is homologous to the tetramerization domain of voltage-gated K+ (T1) channels. Recent studies have unveiled the crucial role of this class of proteins in important and diversified biological processes i.e. in the insurgence and progression of severe human pathologies including cancer, epilepsy, and obesity [15, 17–23]. It has been shown that some members of the family interact with Cul3 and act as substrate adaptors in the CRL3 system [15, 18, 24–28]. Among them, KCTD11 is an important regulator of the Hedgehog pathway since it works as substrate adaptor for HDAC1 [15]. Previously, we reported that a Cul3-based peptide (residues 49–68 hereafter denoted as Cul3^49-68^) is able to bind KCTD11^BTB^, despite its rather low structural content as shown by CD characterization[16]. Here we evaluate the impact of stapling on biochemical/biophysical properties of Cul3^49-68^ derivatives. In particular, we report the design, synthesis, biochemical and structural characterization of novel Cul3-derived peptides obtained through the stapling of different Cul3^49-68^ regions.

## Materials and Methods

### Notation

Previous analyses have shown the coexistence of two distinct forms of KCTD11 [17]. The KCTD11^BTB^ domain considered in the present study corresponds to the folded BTB region of the long form (isoform 2 of the UniProt Code Q693B1-2). Accordingly, the numbering of KCTD11^BTB^ residues used in the text follows this form. Residues of Cul3-derived peptides have been numbered according to their location in Cul3 sequence.

### Reagents

TFA, scavengers, DCM, FITC isothiocianate*N*-(+)-biotinyl-6-aminocaproic acid, Grubbs catalyst 1st generation were purchased from Sigma-Aldrich; Novasyn TGR resin, coupling reagents and all amino acids were from Novabiochem. DIPEA was from Romil; piperidine from Biosolve; Fmoc-(S)-2-(4’-pentenyl)alanine was from Boc Science.

### Molecular modeling and dynamics

A three-dimensional model of the tetrameric BTB domain of KCTD11^BTB^ (residues 15–116) was generated using standard molecular modeling techniques as implemented in the Swiss Model server (http://swissmodel.expasy.org/) coupled with manual intervention (see supplementary material for details). The crystallographic models of (a) the structure of KCTD5^BTB^ pentamer [23] and (b) the structure of the BTB domain of Akv3.1 voltage-gated potassium channel [29] were used as templates (see supplementary material for details and S1 Fig. for sequence alignments). The complex between the Cul3-based peptide (residues 49–68 of Cul3) and KCTD11^BTB^ was generated by using the structure of the complex between the BTB-containing protein SPOP^BTB^ and Cul3 (PDB code 4EOZ) as template. The final complex (KCTD11^BTB^-Cul3^49-68^)_4_ was generated by docking the peptide in the four equivalent binding pockets of KCTD11^BTB^ tetramer.

The energy of this KCTD11^BTB^ model was minimized by using GROMACS software package 4.5.5. GROMACS package was used in the subsequent simulation carried out using (KCTD11^BTB^-Cul3^49-68^)_4_ as starting structure. Details about the parameters and the protocol adopted are reported in the supplementary material. To evaluate whether the simulation reached an adequate convergence in the essential space [30], the root mean square inner product (RMSIP) between two halves of the equilibrated trajectory was calculated as described by Di Nola and co-workers [31, 32]. The RMSIP between the first 10 eigenvectors is defined as

$$\sqrt{\frac{1}{10}\overset{10}{\underset{i=1}{\sum }}\overset{10}{\underset{j=1}{\sum }}{\left(\eta_{i}^{a}\eta_{j}^{b}\right)}^{2}}$$

Where $\eta_{i}^{a}$ and ${\text{η}}_{j}^{b}$ are the *i* ^th^ and *j* ^th^ eigenvectors from the first and second half of the equilibrated trajectory, respectively.

### Peptides synthesis and purification

Cul3^49-68^, Cul3^49-68EN^, Cul3^49-68LA^, Cul3^49-68SL^ and Cul3^49-68AA^ peptides were obtained on solid phase by Fmoc strategy. To mimic the charge status of the fragment within the parent protein, the *N*- and *C*-termini were acetylated and amidated, respectively. The syntheses were carried out with Novasyn TGR resin (substitution 0.25 mmol g^-1^), using standard Fmoc amino acids and Fmoc-(S)-2-(4’-pentenyl) alanine. Coupling reactions for standard residues were carried out by using 10 equivalents (eq) of Fmoc protected amino acids activated *in situ* with HBTU (9.8 eq)/HOBt (9.8 eq)/DIPEA (20 eq) in DMF for 1 h. Fmoc-(S)-2-(4’-pentenyl)alanine residues were coupled by using 2.5 eq of amino acids and COMU (2.5 eq)/DIPEA (5 eq) in DMF for 6 h.

Fmoc deprotection was performed with 30% piperidine in DMF (10 x 2 min).

Before the cleavage from the resin, the peptides were acetylated, biotinylated or labeled by FITC at *N*-terminus to obtain the corresponding derivatives. The acetylation reaction was carried out using a solution of acetic anhydride (0.5 M)/DIPEA (0.015 M)/HOBt (0.125 M) in DMF (4.7: 4: 91.3 v/v/v) (2 x 15 min). Biotinylated peptides were obtained using a solution of *N*-(+)-biotinyl-6-aminocaproic acid (2 eq)/PyBop (2 eq)/DIPEA (4 eq) in DMF (overnight).

FITC labelling reaction were performed with FITC isothiocianate (2 eq)/ DIPEA 4 (eq) in DMF (overnight).

For ring-closing metathesis, 25 μmol of resin-bound peptide was added to a solution of 20 mM of Grubbs catalyst 1st generation in DCM dry under argon at room temperature (2 x 2 h). The reaction was monitored by ESI-LC-MS instrument, (ThermoFinnigan, NY, USA) equipped with a diode array detector combined with an electrospray ion source and ion trap mass analyzer, using a Phenomenex Jupiter Proteo column (150 × 2 mm; 4 μm; 90 Å) and a linear gradient of H_2_O (0.1% TFA)/CH_3_CN (0.1% TFA) from 20 to 80% of CH_3_CN (0.1% TFA) in 15 min at flow rate of 200 μL/min.

All peptides were cleaved off the resin by treatment with a mixture of TFA/H_2_O/EDT/TIS (94:2.5:2.5:1 *v/v/v/v*) for 3 h at room temperature. The resins were filtered and the crude peptides were precipitated with diethyl ether, dissolved in H_2_O/CH_3_CN mixture (1: 1 *v/v*) and lyophilized. The peptides were purified by preparative RP-HPLC on the Shimadzu system equipped with a UV-Vis detector SPD10A using a Phenomenex Jupiter Proteo column (21.2 × 250 mm; 4 μm; 90 Å) and a linear gradient of H_2_O (0.1% TFA)/CH_3_CN (0.1% TFA) from 20 to 80% of CH_3_CN (0.1% TFA) in 30 min at flow rate of 20 mL/min. The collected fractions containing the desired compounds were lyophilized. The identity and purity of all peptides were assessed by LC-MS. The final yields of Cul3^49-68^ and stapled peptides were 25%. Fluorescent and biotinylated derivatives were obtained with a final yield of about 20%.

### Protein expression and purification

KCTD11^BTB^ was expressed as a recombinant fusion protein with thioredoxin A (TrxA). The expression and purification were performed as previously described [17]. The protein, extensively dialysed against 20 mM phosphate buffer pH 7.5, 150 mM NaCl, was used for different purposes. The pET28/KCTD5^BTB^ plasmid was a gift of Prof. Goldstein (University of Chicago). KCTD5^BTB^, corresponding to the folded BTB domain of KCTD5 (residues 44–145), was expressed and purified as elsewhere reported [33, 34].

### Circular dichroism spectroscopy studies

Far-UV CD spectra were collected on a Jasco J-810 spectropolarimeter from 190 to 260 nm at 25° C using a quartz cell with of 0.1-cm path length. The spectra were acquired using the following parameters: 0.5 nm step resolution, 10 nm/min scanning speed, 8s response time and 1 nm spectral band width. The spectra were recorded at peptide concentration ranging from 10 to 90 μM, at pH 3.0 (0,1% TFA) and at pH 7.0 (10 mM phosphate buffer). The final spectra were expressed as molar ellipticity θ (deg cm^2^ dmol^-1^) *per* residue. Deconvolutions of CD spectra were obtained using the CONTINLL program, which is part of the web-based program CdPro (http://lamar.colostate.edu/~sreeram/CDPro/).

### ELISA assay

For ELISA assays, 5 μg/mL streptavidin in phosphate/citrate buffer pH 5.0 was incubated overnight at 37°C for coating. Firstly, wells were coated with 0.8 μM biotinylated Cul3^49-68^ peptides in phosphate buffered saline (PBS) 1X for 1 h at room temperature. Binding step was performed with increasing concentrations (0.4, 0.75, 1.5, 3.8, 7.6 and 15.6 μM) of His-TrxA- KCTD11^BTB^ in PBS 1X. His-TrxA was used as negative control in the same concentrations. As blocking solution 3% gelatine, 0.05% Tween-20 in PBS 1X was used for 1 h at 37°C. Washes were executed with PBS 1X. To reveal the occurred interaction mouse anti-His monoclonal antibody was incubated in 1:1000 dilution at room temperature for 2 h; then, horseradish peroxidase-conjugated anti-mouse antibody (Pierce) was diluted 1:10000 in 1% gelatin, PBS 1X and incubated at 20°C for 1 h. The colorimetric reaction has been carried out with SIGMAFAST OPD reagent (Sigma Aldrich), according to the manufacturer’s instructions. Finally, a Model 680 Microplate Reader (Bio-Rad, Hercules, CA-USA) has been used for readings at 490 nm; data were processed by a Microplate Manager 5.2 program. The reported data are mean values of triplicate experiments.

### Fluorescence polarization

Polarization measurements were conducted using a Synergy 2 Multi-Mode Microplate Reader (BioTek) with the fluorescence polarization module. Excitation wavelength was 460 ± 40 nm and emission was detected at 528 ± 20 nm. For each independent experiment a concentration of 2 μM of FITC- Cul3^49-68^ peptides was used. The final volume per well in 384-well plate format was 60 μl. Binding assays were performed adding KCTD11^BTB^ at increasing concentration (from 0.1 μM to 15 μM) [35]. Analogous experiments were conducted on KCTD5^BTB^,expressed and purified as previously described [33]. Binding curves were generated and dissociation constants (K_D_) were calculated from the nonlinear regression curve using GraphPad Prism and the following equation [35]:

$$FP-FP_{0}=B_{MAX}*\frac{\left[KCTD11^{BTB}\right]}{\left(K_{D}+\left[KCTD11^{BTB}\right]\right)}$$

in which FP is the fluorescence polarization (measured in mP), [KCTD11^BTB^] is the molar concentration of KCTD11^BTB^ as monomer, Bmax is the maximum polarization, and FPzero is a correction factor to account for the intrinsic polarization of the peptide evaluated in each experiment. A competition experiment with the unlabeled peptide (Cul3^49-68LA^) was performed.

### Nuclear magnetic resonance spectroscopy

NMR samples of Cul3^49-68^, Cul3^49-68EN^, Cul3^49-68LA^ were dissolved in 500 μl of a water solution containing 0.1% TFA and 50 μl of D_2_O adjusted at pH = 3. All of the NMR experiments were acquired on a Varian Unity INOVA 500 MHz spectrometer.

A standard set of 2D experiments, TOCSY [36] and NOESY [37] were acquired at 25°C. The TOCSY experiments were recorded using a mixing time of 70 ms while the NOESY spectra were carried out with a mixing time of 300 ms. Chemical shifts were referenced to external TMS (tetramethylsilane) (δ = 0 ppm).

NMR data were processed by using Varian (VNMR6.1B) software and analyzed and assigned using XEASY program [38]. Chemical shift deviations of H^α^ protons for Cul3^49-68^, Cul3^49-68EN^ and Cul3^49-68LA^ were estimated by using as references random coil H^α^ chemical shift values [39].

The structure of the peptides were calculated using CYANA [40]. The upper limits of the distance restraints were obtained from the NOESY cross-peak intensities using the calibration routine of CYANA. During the CYANA runs, the peptides were calculated with and without the olefinic linker, and the non-standard amino acid (S)-2-(4’-pentenyl) alanine was added in the cyana library. The structures were analyzed and visualized by using the program MOLMOL [41] and PROCHECK [42].

The dihedral angles were estimated using HN and Hα chemical shifts by the software PREDITOR[43]. The secondary structure propensity has been evaluated based on the HN and Hα chemical shifts using the software Secondary Structure Propensity (SSP)[44].

### Serum stability

Human serum (Sigma Aldrich-Italy) was centrifuged at 13000 rpm for 10 min to remove lipids;the supernatant was collected and incubated at 37°C for at least 15 min. The assay started upon the addition of the peptides to the 25% serum for a final peptide concentration of 0.3 μM.

200 μL aliquots of the samples were taken after the following time points: 0, 60, 120, 180, 360 and 900 min. The aliquots were mixed with 100 μL of 15% TFA and incubated at 4°C for at least 15 min to induce the precipitation of the serum proteins. The supernatants were collected for each sample after centrifugation at 13000 rpm for 10 min and stored at -20°C.

The serum stability of peptides was followed by LC-MS analysis. using a Phenomenex Jupiter C4 (150 × 2 mm; 5 μm; 300 Å) and a method consisting of an isocratic step with H2O (0.1% TFA)/CH3CN (0.1% TFA) (70:30) for 10 min and a linear gradient of H_2_O (0.1% TFA)/CH_3_CN (0.1% TFA) from 30 to 80% of CH_3_CN (0.1% TFA) in 20 min at flow rate of 200 μL/min.

The assays were performed in triplicate.

## Results

### KCTD11—Cul3 recognition and peptide design

**Fig 1:**
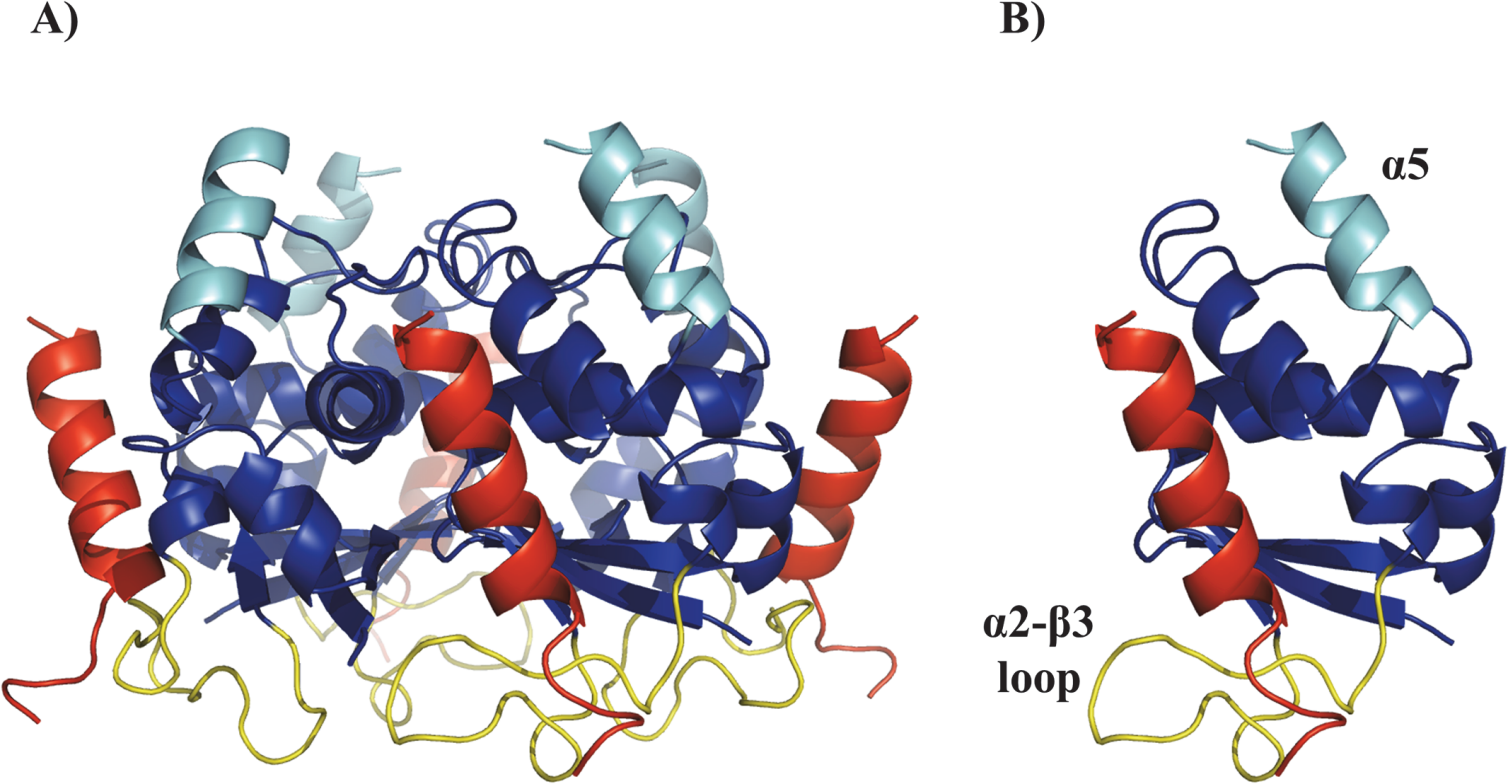
Three-dimensional model of the complex (KCTD11^BTB^-Cul3^49-68^)_4_. (A) The model of the three-dimensional structure of the complex (KCTD11^BTB^-Cul3^49-68^)_4_ that was used as starting model in the molecular dynamics simulation. The peptide Cul3^49-68^ is coloured in red. The C-terminal helices (α5) which protrude toward the solvent and the interacting loop α2-β3 of the four KCTD11^BTB^ chains are shown in cyan and yellow, respectively. (B) A snapshot of a monomer KCTD11^BTB^-Cul3^49-68^ which highlights the location of key elements of the model is reported.

In order to design variants of the peptide with improved Cul3^49-68^ biochemical/biophysical properties, we preliminarily investigated the mechanism of molecular recognition between KCTD11 and Cul3^49-68^. Previous investigations have shown that KCTD11^BTB^ adopts a tetrameric structure [17]. Therefore, we generated a homology model of the KCTD11^BTB^ tetramer based on the structure of two related proteins (i) the voltage-gated potassium channel Akv3.1[29] and (ii) KCTD5 [23] (see materials and methods for details). Notably, the most important residues involved in the interactions formed at Akv3.1 tetramer interface (Asp56 and Arg70) are conserved in KCTD11 sequence (Asp66 and Arg80). Indeed, the inspection of the subunit interface indicates that the electrostatic interactions that stabilize KCTD11^BTB^ tetramer are similar to those found for Akv3.1. The main difference between the model of tetrameric KCTD11^BTB^ and Akv3.1 is the orientation of the α5 helix located at the very C-terminal end of the domain. In the BTB domain of Akv3.1 this helix, which is deeply involved in the coordination of Zn ion, folds against the rest of the protein. In KCTD11^BTB^ the C-terminus of this helix protrudes toward the solvent (Fig. 1). It is likely that the location of α5 promotes the correct relative orientation between KCTD11^BTB^ and the C-terminal substrate-binding domain of the protein, which is involved in the recognition process of HDAC1 [15], whose structure is hitherto unknown.

The model for the tetrameric association between KCTD11^BTB^ and Cul3^49-68^ was generated using the structure of the complex between Cul3 and SPOP^BTB^ as template (see materials and methods for details). The modeling indicates that Cul3^49-68^ binds KCTD11^BTB^ in a cavity located at the tetramer interface (Fig. 1). In order to gain further data about Cul3^49-68^-KCTD11^BTB^ recognition process, the structure of the complex was used as a starting model in molecular dynamics studies.

In line with previous binding experiments [16], the analysis of the (KCTD11^BTB^-Cul3^49-68^)_4_ assembly during the simulation indicates that structures reach a rather stable state in the 30–130 ns interval of the simulation (S2 Fig.). This is evident from the analysis of the RMSD values and the gyration radius. Moreover, the RMSIP value computed on two independent halves of the trajectory was 0.65 (see supplementary material for details).This indicates that an adequate convergence of the simulation was achieved.

**Fig 2:**
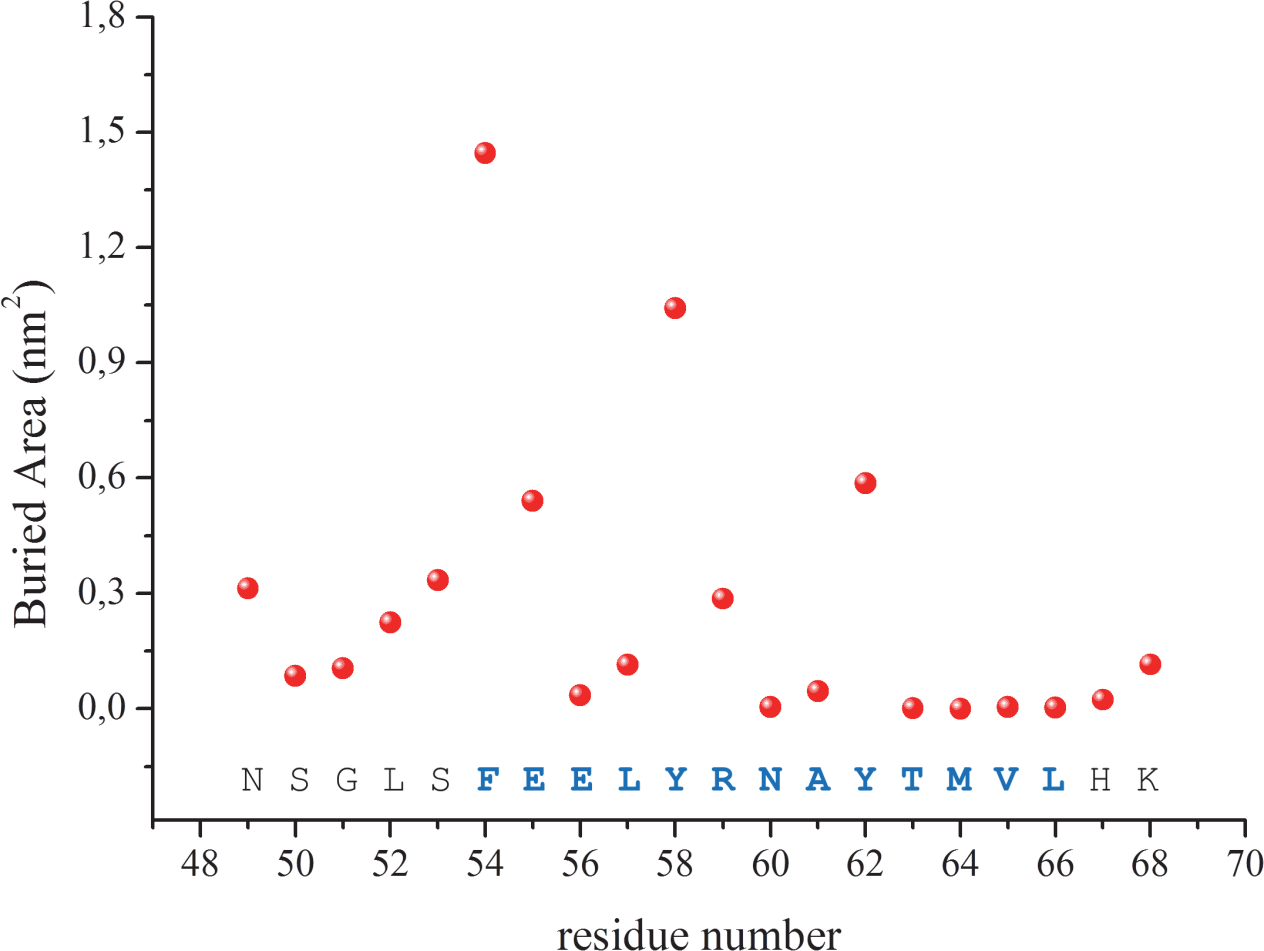
Buried area of Cul3^49-68^ residues upon complex formation with KCTD11^BTB^.

The evaluation of the protein and the peptide dynamics throughout the trajectory, carried out through the analysis of the root mean square fluctuation (RMSF) values calculated on C^α^ atoms in the equilibrated region of the trajectory, suggests that structured regions of KCTD11^BTB^ are rather rigid (RMSF ~1.0 Å) (S3A Fig.). Nevertheless, RMSF values increase up to 4. Å for loop and terminal regions. Very high RMSF values are displayed by the α2-β3 loop, which is believed to play a significant role in BTB Cullin recognition. With the exception of the terminal ends, Cul3^49-68^ also displays rather low RMSF values that often are below 2.0 Å (S3B Fig.). The relative rigidity of Cul3^49-68^ is confirmed by the monitoring of the secondary structure content of the peptide along the trajectory. Indeed, the peptide retains a significant level of secondary structure throughout the simulation, despite some local unfolding observed at the C-terminus of the helix (S4 Fig.). The analysis of the buried area of the peptide residues upon complex formation clearly indicates that aromatic residues are deeply involved in intermolecular interactions (Fig. 2). Indeed, the largest buried areas correspond to the residues Phe54, Tyr58 and Tyr62, which are located on the same side of the helix. This observation is corroborated by the analysis of specific interactions that are preserved in the simulations (S5 Fig.). Most conserved ones are those established by residues of the regions α2-β3 and α4-α5 of KCTD11^BTB^ and the aromatic residues Tyr58 and Tyr62 of Cul3^49-68^. These include both hydrogen bonding and hydrophobic interactions established by aromatic side chains.

**Fig 3:**
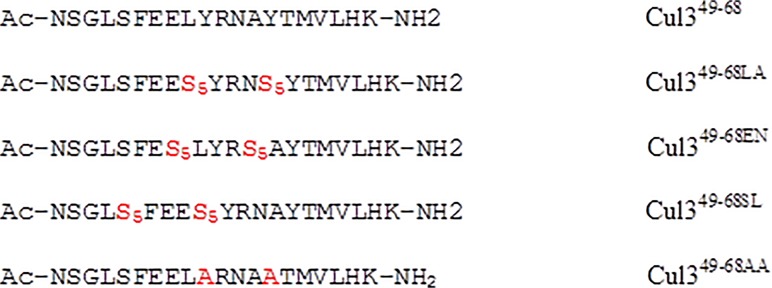
Aminoacid sequences of all peptides. The replaced residues are highlighted in red. S_5_ corresponds to the non-standard amino acid (S)-2-(4’-pentenyl) alanine.

Overall these findings indicate that the helicity of the peptide represents a major requirement for its efficient binding to KCTD11^BTB^. However, solution studies demonstrate that Cul3^49-68^ presents a very low intrinsic propensity to adopt this kind of structure [16]. To evaluate the effect of an increase of the helical propensity of the peptide biochemical/biophysical properties we planned to incorporate an all-hydrocarbon "staple" into this peptide. Since it has been shown that the extent of α-helix stabilization provided by stapling is substantially context dependent [3], we used the results of the MD analysis to design Cul3-derived stapled peptides. The analysis of the buried area of Cul3^49-68^ residues on KCTD11 (Fig. 2) binding suggests that residues Tyr58 and Arg59 are surrounded by completely solvent-exposed residues (Glu56, Leu57, Asn60 and Ala61). In this scenario, staples encompassing one turn of the helix (residues *i* and *i+4*), such as those obtained through the replacement of the pair Glu56-Asn60 or alternatively Leu57-Ala61, were expected to produce minimal perturbation of the binding site between the protein and the peptides. Therefore, we designed two stapled variants of Cul3^49-^68 in which either Glu56-Asn60 (denoted as Cul3^49-68EN^) or Leu57-Ala61 (Cul3^49-68LA^) were replaced with the non-standard amino acid (S)-2-(4’-pentenyl) alanine (S5). Moreover, we also characterized the mutant Cul3^49-68AA^, a non-stapled variant in which Tyr58 and Tyr62 were replaced with Ala residues. Since Phe54 side chain is buried upon complex formation, we also designed a staple by replacing the pair Ser53-Leu57 (Cul3^49-68SL^) with the aim to increase the local helicity of the peptide, although this region is not fully structured in Cul3 where the helix spans from residue 54 to 66. The sequences of the peptides that have been characterized are reported in Fig. 3.

### Circular dichroism characterization of stapled peptides

**Fig 4:**
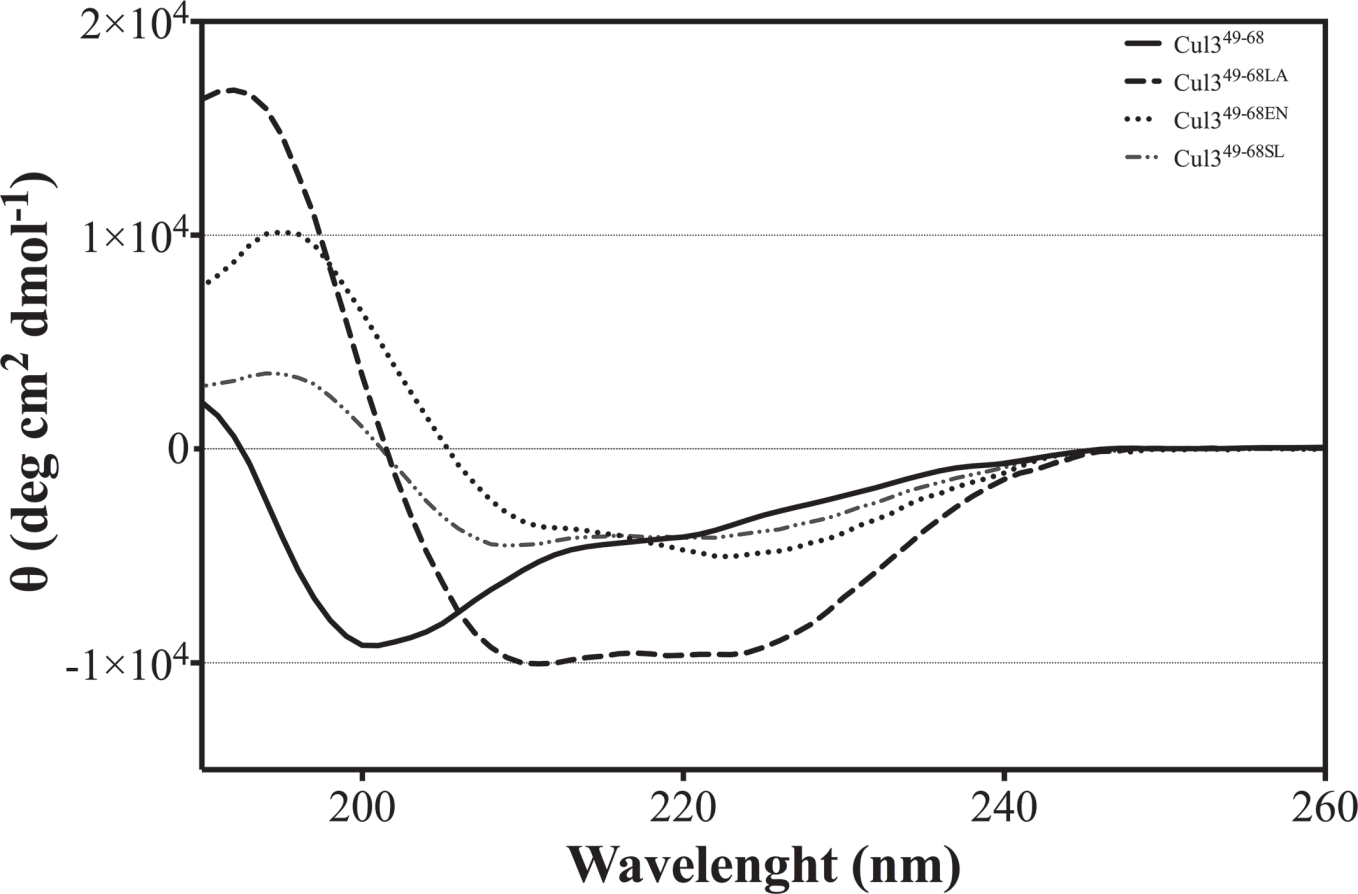
CD spectra. Far-UV CD spectra of Cul3^49-68^, Cul3^49-68EN^, Cul3^49-68LA^, and Cul3^49-68SL^. Spectra were acquired in 10 mM phosphate buffer pH 7.0.

Although detailed structural data on these peptides were obtained by NMR (see below), preliminar insights into the structural preferences of the peptides considered in the present study were gained by far-UV CD experiments. The spectra were registered in 10 mM phosphate buffer at pH 7.0. In line with a previous characterization of the peptide [16], the CD spectrum indicates that Cul3^49-68^ presents a very limited structural content (Fig. 4). Notably, all stapled peptides exhibit an increased amount of helical structure as indicated by the positive peak at wavelength of ~ 190 nm and the two negative peaks at about 210 and 222 nm. The intensity of the positive peak and the location of the negative ones suggest that Cul3^49-68SL^ is endowed with slightly lower secondary structure content. Indeed, the ratios of disordered/helical structural contents were estimated to be 3.98, 1.95, 1.72 and 0.93 for Cul3^49-68^, Cul3^49-68 SL^, Cul3^49-68EN^ and Cul3^49-68 LA^, respectively. This finding confirms that these peptides have different helical propensities. The CD spectra were also collected at pH 3.0 for all peptides. They were roughly identical to those collected at pH 7.0 (S6 Fig.). No concentration effects were observed on the shape and intensity of the spectra in the range (10^-6^-10^-5^ M) taken into consideration.

### Affinity

**Fig 5:**
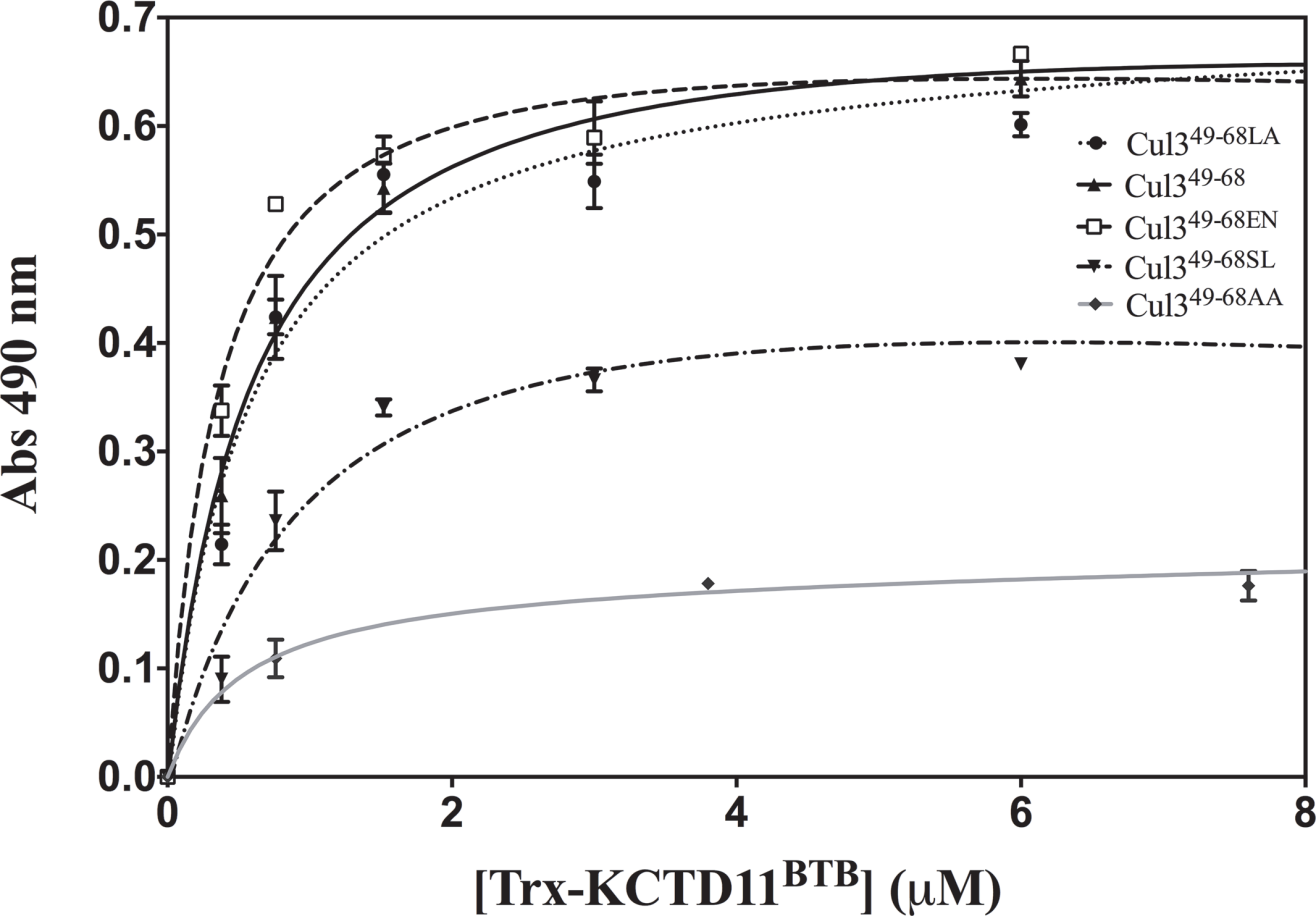
ELISA assays. Binding curves obtained from ELISA on KCTD11^BTB^ using the biotinylated peptides Cul3^49-68^ (▲),Cul3^49-68LA^ (●),Cul3^49-68EN^ (_▀_),Cul3^49-68SL^ (▼) and Cul3^49-68AA^(♦).

**Fig 6:**
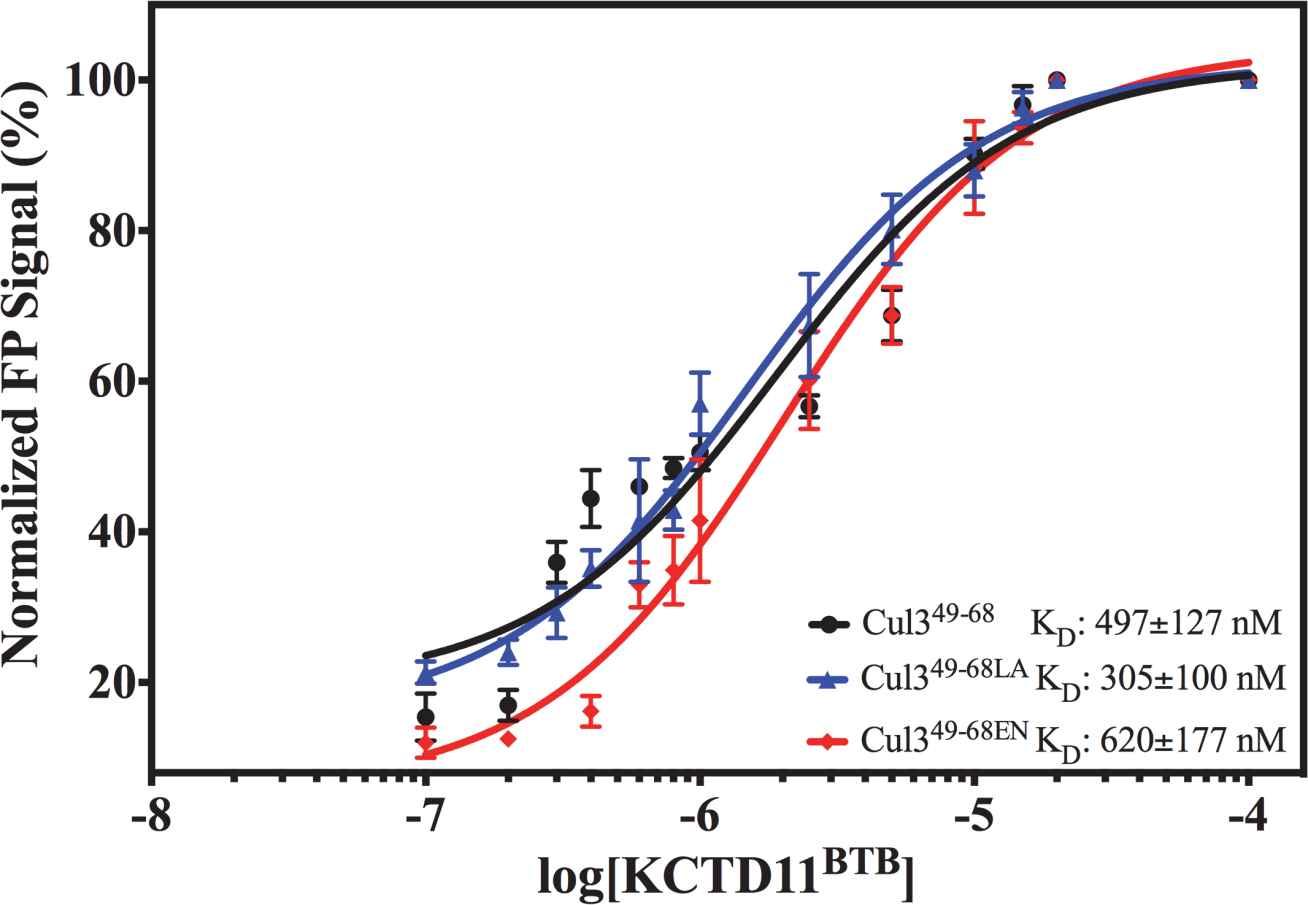
Normalized Fluorescence Polarization data for peptides as a function of [KCTD11^BTB^]. Peptides were plated at a final concentration of 2 μM, and the interaction with KCTD11^BTB^ was tested over a concentration range of 0.1 nM to 20μM. Dissociation constants were calculated using nonlinear regression and are presented as mean ± standard error of triplicates.

The ability of the peptides to bind recombinant KCTD11^BTB^ was qualitatively checked by ELISA (Fig. 5). Cul3^49-68^, Cul3^49-68EN^ and Cul3^49-68LA^ resulted to be able to bind KCTD11^BTB^ with a comparable affinity as highlighted by the trend of ELISA assays (Fig. 5). On the other hand, Cul3^49-68SL^ showed a greatly reduced affinity. No relevant binding was detected for Cul3^49-68AA^. To obtain a quantitative estimate of the dissociation constants of the most affine binders, Cul3^49-68^, Cul3^49-68EN^ and Cul3^49-68LA^, fluorescence polarization experiments were carried out (Fig. 6 and S7 Fig.). The fitting of data gave the following K_D_ values: 497 ± 127 nM, 620 ±177 nM and 305 ± 100 nM for Cul3^49-68^, Cul3^49-68EN^ and Cul3^49-68LA^, respectively. A possible contribution of the fluorophore FITC to the binding to KCTD11^BTB^ was excluded by a competition experiment with unlabeled Cul3^49-68LA^ (S8 Fig.).

**Fig 7:**
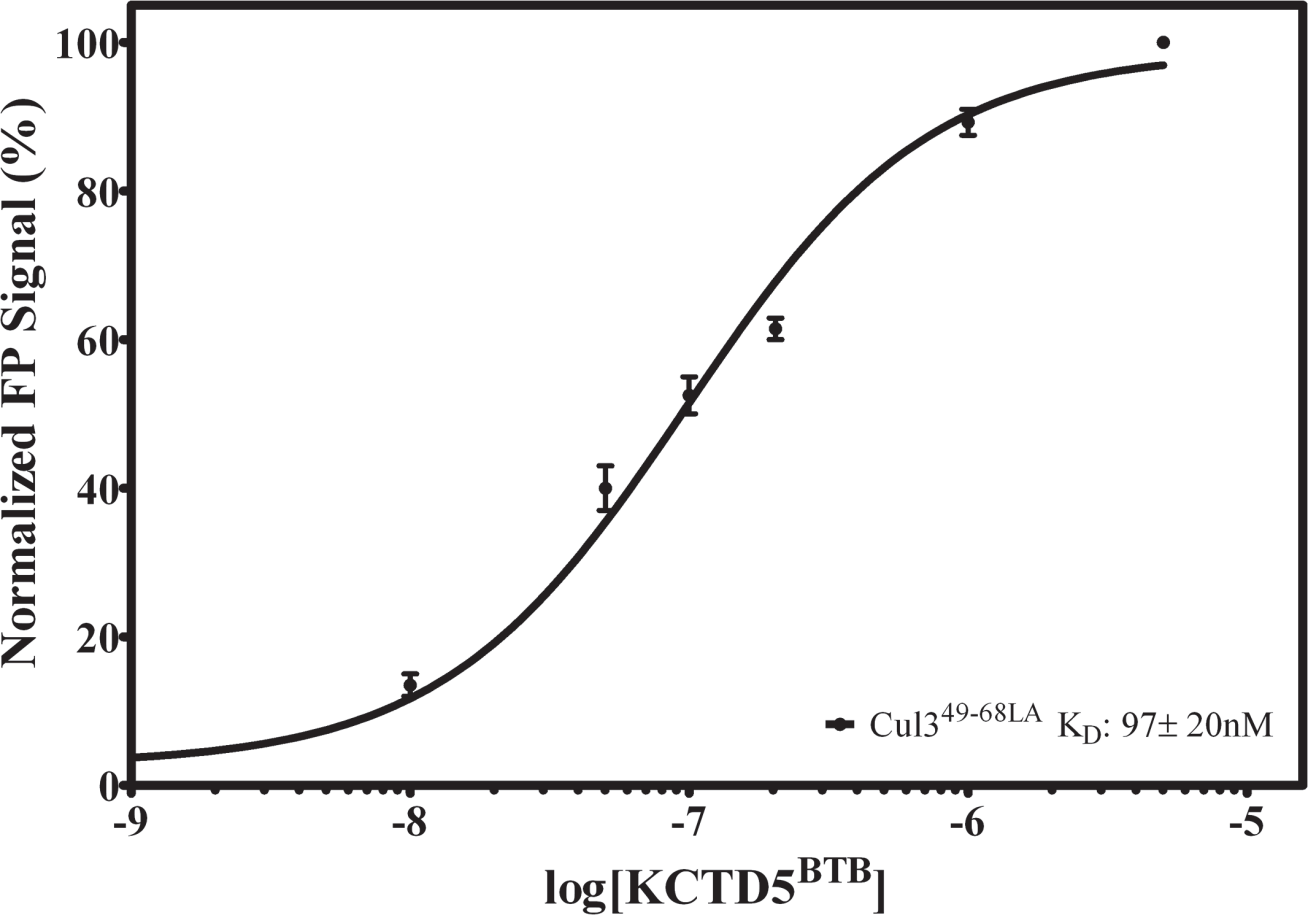
Normalized Fluorescence Polarization data for Cul3^49-68LA^ as a function of [KCTD5^BTB^]. Peptide was plated at a final concentration of 1 μM, and the interaction with KCTD5^BTB^ was tested over a concentration range of 0.1 nM to 5 μM. Dissociation constants were calculated using nonlinear regression and are presented as mean ± standard error of triplicates.

Moreover, the fluorescence polarization experiment was also extended to the pentameric KCTD5^BTB^. This experiment demonstrates that Cul3^49-68LA^ is able to interact also with this domain (97±20nM) (Fig. 7). Interestingly, Cul3^49-68LA^ recognizes BTB domains endowed with different oligomeric organization with a high affinity.

### NMR structure

**Fig 8:**
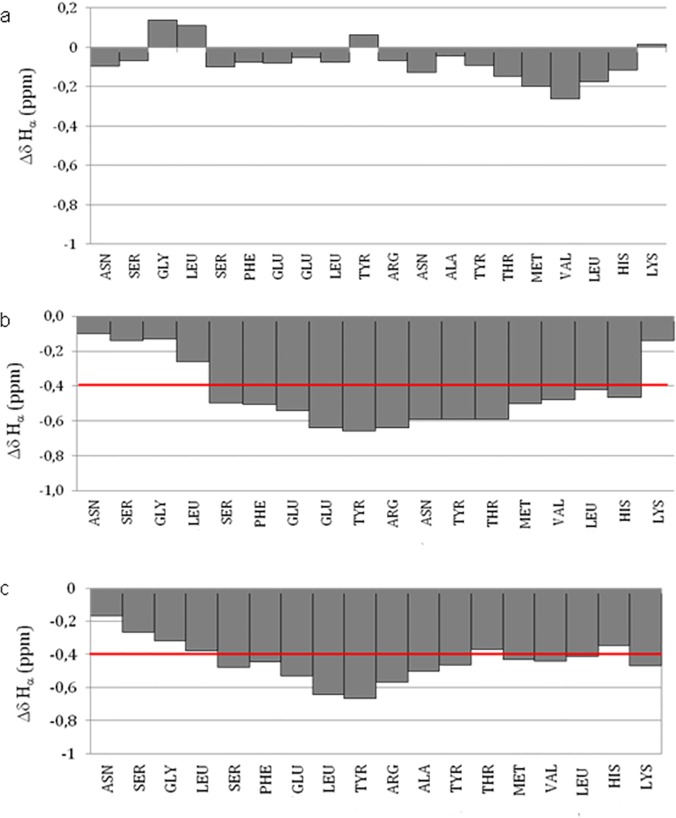
Hα secondary chemical shift for Cul3^49-68^ (a), Cul3^49-68LA^ (b) and Cul3^49-68EN^ (c) peptides. The red line indicates the average upfield shifts observed in peptides database for α-helix.

NMR measurements for the Cul3^49-68^ peptide have been carried out at pH 3.0. These conditions have been chosen as this peptide has shown a tendency to aggregate at the NMR concentrations upon increasing the pH value. The complete assignment of the ^1^H resonances has been obtained (for chemical shifts see S1 Table). The narrow range of the HN resonances dispersion and the absence of secondary structure diagnostic NOEs clearly indicate that Cul3^49-68^ does not assume a definite conformation in these conditions, in agreement with the far-UV CD results. Nevertheless, due to their sensitivity to the chemical environment, the deviations of the Hα chemical shifts with respect to their random coil values (Fig. 8) demonstrate a slight tendency of the peptide to assume a helical conformation, reflecting the helical nature of the sequence 54−66 in the parent protein.

The NMR spectra for the stapled peptides (Cul3^49-68EN^ and Cul3^49-68LA^) have been carried out in the same conditions obtaining the complete assignment of their resonances. As expected, the HN resonances dispersion and the analysis of the observed Hα chemical shift deviations for both stapled peptides indicate that the incorporation of two (S)-2-(4’-pentenyl)alanine modified amino-acids into the Cul3^49-68^ sequence results in a net increase of α-helical content (S2 and S3 Tables). On the contrary, in the case of Cul3^49-68SL^ peptide, the NMR spectrum indicates that the introduction of the stapling bridge in these positions does not significantly improve the helical content (S9 Fig.).

**Fig 9:**
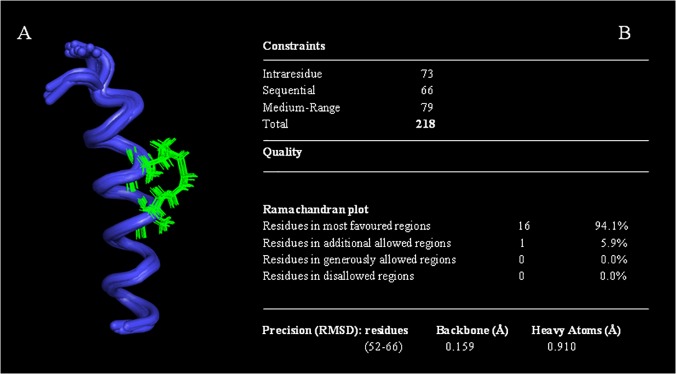
The NMR structure of Cul3^49-68EN^. (A) NMR ensemble of the best 20 structures of Cul3^49-68EN^ peptide. In the figure the *i*, *i + 4* staple bridge produced by the combination of cross-linking (S)-2-(2’-pentenyl) alanine is shown in green. (B): Structural statistics for the Cul3^49-68EN^ peptide.

**Fig 10:**
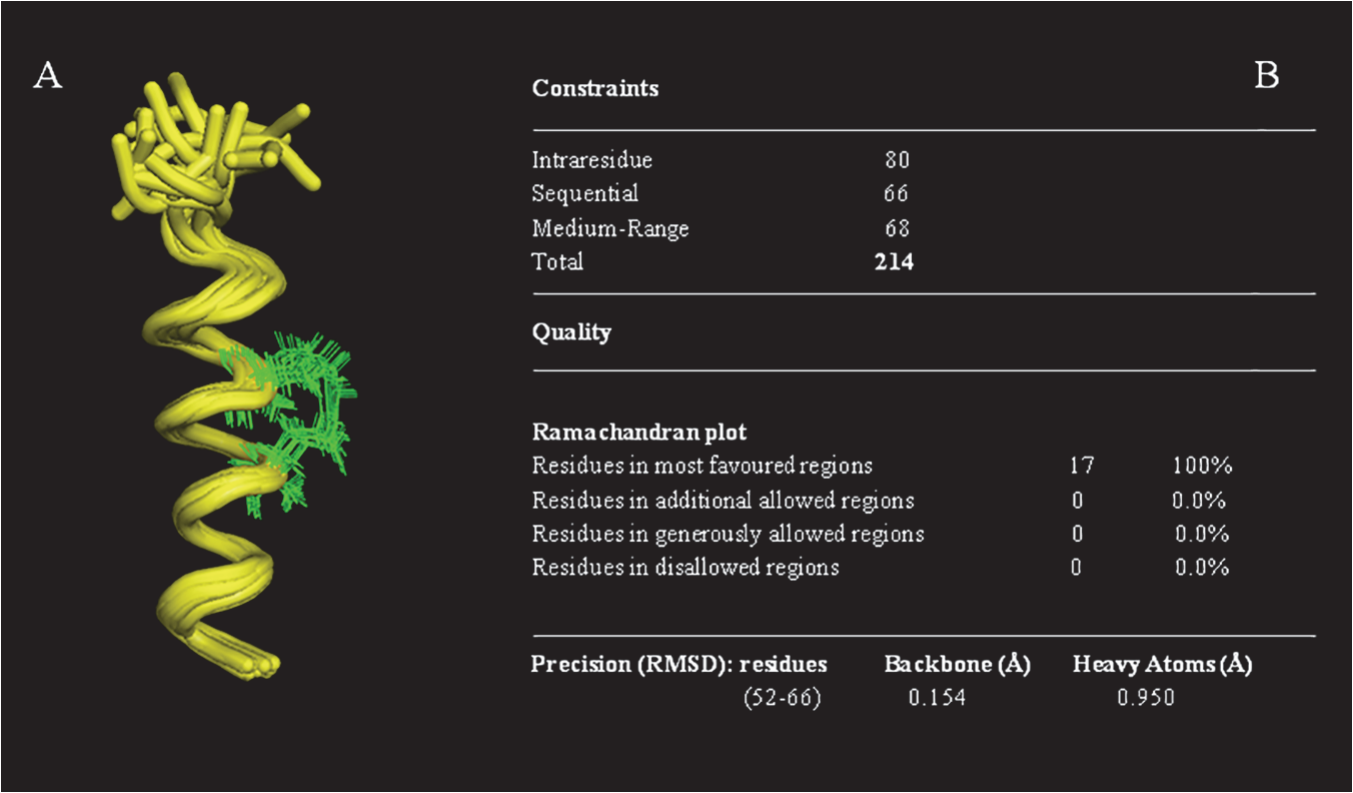
The NMR structure of Cul3^49-68LA^. (A) NMR ensemble of the best 20 structures of Cul3^49-68LA^ peptide. In the figure the *i*, *i + 4* staple bridge produced by the combination of cross-linking (S)-2-(2’-pentenyl) alanine is shown in green. (B) Structural statistics for the Cul3^49-68LA^ peptide.

The good quality of the spectra and the good number of NOE cross peaks encouraged us to calculate the structure of Cul3^49-68EN^ and Cul3^49-68LA^ peptides. At first, we predicted the secondary structure content of both peptides based on the Hα and HN chemical shifts using the Chemical Shift Index method [45]. Moreover, the helical content was further confirmed by the analysis of the predicted φ and ψ angles (S4 and S5 Tables) and the secondary structure propensity for each residue of both peptides was also evaluated (S10A and B Fig.). The structures were calculated based on 218 and 214 experimental NOE derived distance constraints respectively (Figs. 9 and 10).

The ensemble of the 20 best structures of Cul3^49-68EN^ and Cul3^49-68LA^ show a backbone r.m.s.d. of 0.159Å (residues 52–66) and 0.154 Å (residues 52–66) and an average target function of 0.84 ± 0.03 and 0.63 ± 0.02 respectively. There were no distance violations greater than 0.2 Å and no dihedral violations greater than 5°. The structural statistics of the 20 best structures for the two stapled peptides are reported in Figs. 9 and 10.

Both Cul3^49-68EN^ and Cul3^49-68LA^ peptides adopt an extensive helical conformation (Figs. 9 and 10) that allows the three aromatic residues playing a crucial role in the recognition mechanism of these peptides (Phe54, Tyr58 and Tyr62) residing in the same face of the helix. The two structures are however slightly different. In particular, Cul3^49-68LA^ α-helix encompasses residues Leu52-Val65 and appears longer at the N-terminus than the Cul3^49-68EN^ helix which is in turn longer at the C terminus spanning residues Phe54-His67. On the contrary, the overlay of a diagnostic portion of the NOESY spectrum of Cul3^49-68SL^ with the same region of Cul3^49-68EN^ spectrum shows the absence of the typical α-helix NOE connectivities in Cul3^49-68SL^spectrum (S11 Fig.).

### Serum stability of stapled peptides

The serum stability of the stapled peptides was monitored by LC/MS. It was found that the half-life of Cul3^49-68^ in serum was 7h similarly to Cul3^49-68SL^ whereas the half-lives of Cul3^49-68EN^ and Cul3^49-68LA^ increased to over 22h under the same conditions (Table 1). Mass spectra analysis indicates a preferential cleavage site at Tyr58 suggesting the presence of a chymotrypsin-like enzyme in the serum medium. Evidently, the presence of the hydrocarbon bridge reduces the accessibility of this residue in Cul3^49-68EN^ and Cul3^49-68LA^ compared to Cul3^49-68^. On the other hand, the stapling between residues 53–57, as in Cul3^49-68SL^, does not affect the accessibility of Tyr58. This observation is consistent with literature data reporting that the stapled peptides can be endowed with increased serum stability compared to the unmodified analogues [2].

**Table 1:** Serum stability of the peptides and assignment of the fragments derived from proteolysis by LC/MS analysis.

**PEPTIDE**	**ESTIMATE *t***_*1/2*_	**Fragment Mass (Da)**	**ASSIGNMENT**
**Cul3** ^**49-68**^	**~7h**	**1075**	**NAYTMVLHK-NH**_**2**_
		**1355**	**Ac-NSGLSFEELYR**
		**1199**	**Ac-NSGLSFEELY**
**Cul3** ^**49-68LA**^	**~22h**		**n.d.**
**Cul3** ^**49-68EN**^	**~22h**		**n.d.**
**Cul3** ^**49-68SL**^	**~7h**	**1075**	**NAYTMVLHK-NH**_**2**_

## Discussion

**Fig 11:**
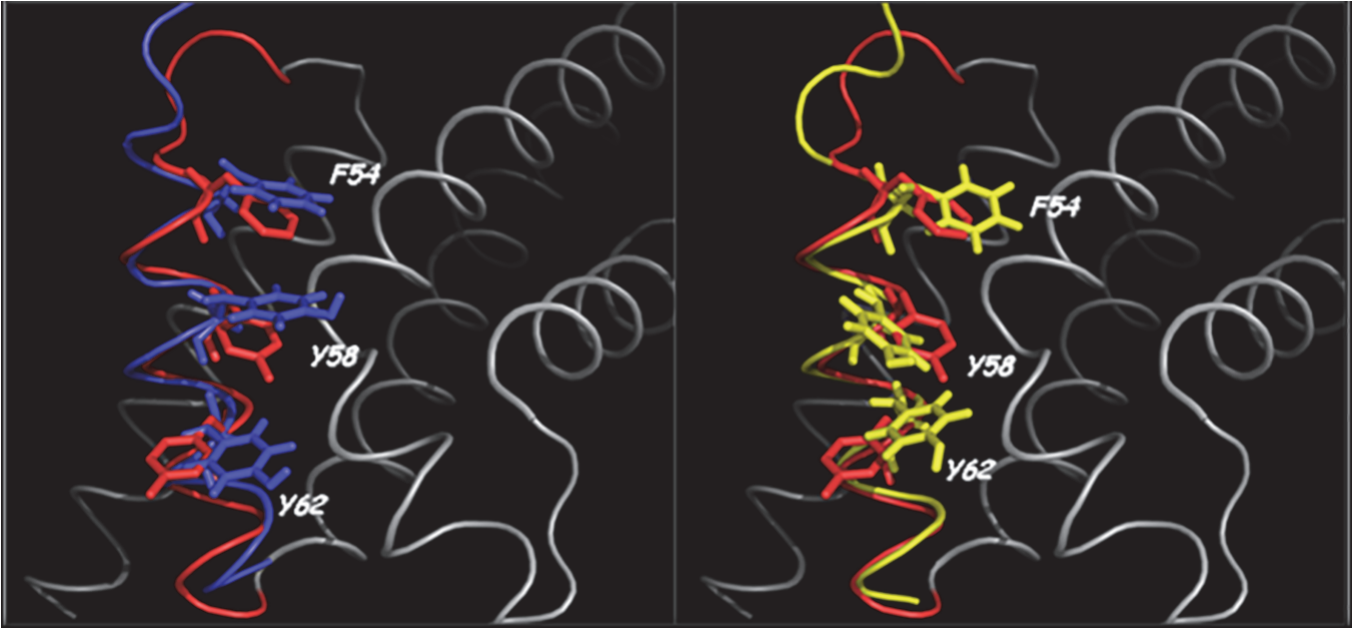
Superposition of the stapled peptides with Cul3. Superposition of the most representative conformer of Cul3^49-68EN^ (left panel; blue) and Cul3^49-68LA^ (right panel; yellow) respectively with the region (in red) encompassing residues Phe54-Leu66 of the crystal structure of Cul3 (PDB code 4EOZ).

Cul3 is a large protein that is able to interact with a variety of different biological partners. The C-terminal region of the protein recruits, *via* the ring protein Rbx1, the E2 enzyme, whereas its N-terminal end anchors BTB-containing proteins that bring the substrate proteins to be ubiquitinated. Intriguingly, BTB-containing proteins that interact with Cul3 often present quite distinct features in terms of both sequence and oligomeric organization. Although other members of the cullin family have been the object of several structural characterizations, the first crystallographic studies on Cul3 have been reported only very recently [12]. These studies have provided interesting insights into the recognition mechanism of Cul3 by some dimeric BTB-containing proteins. In the last few years, an increasing attention has been devoted to an emerging class of potential Cul3-interacting proteins denoted as KCTDs, which are endowed with a larger structural complexity being able to associate in either pentameric or tetrameric states [46]. By combining experimental and theoretical approaches we have recently shown that the molecular recognition between KCTD5 and Cul3 involves a large surface area made of distinct hot spot regions located in the two proteins [34]. Nevertheless, in a preliminary study [16], we showed that a Cul3-based peptide, which comprises the fragment 49–68 of the protein, was able to bind two members of the family (a) the pentameric KCTD5 and (b) the tetrameric KCTD11. However, as shown in the present study, the use of this peptide as a biochemical tool or as a potential lead compound in therapeutic applications is seriously hampered by its limited stability in serum being highly susceptible to protease degradations. In order to improve the biochemical properties of Cul3-based peptides we designed, synthesized and characterized some stapled variants of the peptide [47]. In particular, MD simulations on the complex Cul3^49-68^-KCTD11^BTB^ have highlighted that three aromatic residues (Phe54, Tyr58 and Tyr62) of the Cul3-derived peptide play a major role in KCTD11 recognition. These predictions have been corroborated by the observation that the peptide Cul3^49-68AA^, in which Tyr58 and Tyr62 are replaced by Ala residues, is completely unable to bind KCTD11^BTB^ (Fig. 5). This finding corroborates and extends previous observations obtained by replacing these two Tyr with charged Lys residues [16]. Stapled peptides were therefore designed to make the local region of Phe54 or that of Tyr58 and Tyr62 more structured. The characterization of these variants clearly indicates that the impact of the stapling on the peptide structure and biochemical properties strongly depend on its location. Indeed, the stapling of the residues that are close to Phe54 (peptide Cul3^49-68SL^) produces a very limited increase of the helical content (S10 Fig.). This observation may be explained by considering that the stapled region of Cul3^49-68SL^ is located in the Cul3 structure at the very N-terminus of the helix 54–66. Therefore, this region is intrinsically less prone to adopt a helical state. The significant decrease of Cul3^49-68SL^ affinity for KCTD11^BTB^ compared to the wild-type peptide indicates that the insertion of the stapling in this region likely perturbs the interactions of the peptide with the protein and that this perturbation is not compensated by an increase of the helical content of the molecule. On the other hand, the stapling of the central region of the peptide has a different impact on the properties of Cul3^49-68^. In particular, both Cul3^49-68LA^ and Cul3^49-68EN^ adopt well-defined, although slightly different, helical structures. Indeed, in Cul3^49-68LA^ the α-helix encompasses the residues Leu52-Val65 whereas the one formed by Cul3^49-68EN^ spans residues Phe54 to His67. The comparison of these helices with the helical region 54–66 of Cul3 clearly indicates that both are able to mimic the structure adopted by this region in the parent protein (Fig. 11). The superimposition of the helical region in Cul3 structure (PDB code 4EOZ) on Cul3^49-68EN^ and Cul3^49-68LA^ structures yields RMSD values, computed on the C^α^ atoms, of 1.10 and 0.83 Å, respectively. In addition, the relative orientation of the two interacting tyrosines (residues 58 and 62) with respect to the stapling bridge is, as expected, different in the two structures. These results indicate that the structure of Cul3^49-68LA^ more closely resembles the structure found in Cul3. Although the two peptides both present affinities for KCTD11^BTB^ falling in the sub-micromolar range, some small differences have been detected. In particular, Cul3^49-68LA^, whose structure is more similar to that exhibited by the helix in the parent protein, shows a slight increase in the affinity for KCTD11^BTB^ compared to the wild-type peptide. On the other hand, the stapling introduced in Cul3^49-68EN^ marginally reduced the affinity for KCTD11^BTB^. These findings indicate that the affinity of the stapled peptides limitedly correlates with their structural similarity with the helical region 54–66 of Cul3. Moreover, they also suggest that the increase of helical content does not necessarily reflect into increased affinity for KCTD11^BTB^, as the hydrocarbon bridge of the stapling may perturb the proper protein-protein recognition mechanism.

The insertion of the hydrocarbon bridge may lead to a significant increase of serum stability of these Cul3-derived peptides as both Cul3^49-68LA^ and Cul3^49-68EN^ display a higher resistance to degradation compared to the wild-type peptide. As for the affinity, the location of the stapling is essential for the peptide stabilization since present data show that Cul3^49-68SL^ displays a serum stability comparable to the unmodified peptide. These findings suggest that the insertion of the stapling in the central region of Cul3^49-68^ has a major impact on its biochemical and structural properties. It is important to note that the affinity displayed by Cul3^49-68LA^ for KCTD11^BTB^ and for KCTD5^BTB^ is of the same order of magnitude of those reported for the interaction of Cul3 with some BTB containing proteins. Indeed, Cul3^49-68LA^_-_ KCTD11^BTB^ and Cul3^49-68LA^_-_ KCTD5^BTB^ affinities are intermediate between those exhibited by Cul3 for the BTB domain of SPOP [12] or of KCTD5 [34].

These considerations suggest that stapled peptides efficiently mimic these protein-protein interactions, although BTB-Cul3 recognition involves large and often non-contiguous, surfaces [48]. Therefore, taking also into account their serum stability, the stapled peptides we characterized are potentially able to modulate fundamental biological processes that involve a promiscuous and key protein such as Cul3. Notwithstanding the high performance of the stapled peptides, a further improvement in terms of solubility would be required for their applicability in clinical and experimental setups.

## Supporting Information

S1 File Generation of the KCTD11^BTB^ three-dimensional model; Protocol of the molecular dynamics simulation; Monitoring of the structure stability of the models along the trajectory. (DOCX)

S1 Fig Multiple sequence alignment of the BTB domains of different proteins. α-Helices and β-strands are denoted in blue and red, respectively.
(TIF)

S2 Fig Evolution of (KCTD11^BTB^-Cul3^49-68^)_4_ trajectory structures in simulation. (A) Root mean square deviations, computed using C^α^ atoms, compared to the starting model and (B) gyration radius.
(TIF)

S3 Fig Root mean square fluctuations per residue of KCTD11^BTB^ and Cul3^49-68^ in the equilibrated region of the trajectory. The lines reported in panels A represent residues in α-helices (cyan) or in β-sheets (red). In panel B, the helical residues of Cul3^49-68^ are highlighted in cyan.
(TIF)

S4 Fig Evolution of secondary structure in Cul3^49-68^ in the simulation. Regions in blue, grey, yellow and green represent α-helices, 3–10 helices, turns and bends, respectively.
(TIF)

S5 Fig Preserved intermolecular interactions along the trajectory. Examples of H-bonds and aromatic residues clustering are reported in panels A and B, respectively.
(TIF)

S6 Fig Far-UV CD spectra of Cul3^49-68^, Cul3^49-68EN^, Cul3^49-68LA^, and Cul3^49-68SL^. Spectra were acquired in 0,1% TFA (pH 3.0).
(TIFF)

S7 Fig Dependence of Polarization signal of FITC-peptides as a function of [KCTD11^BTB^]. (TIF)

S8 Fig Dependence of Polarization signal of FITC-Cul3^49-68LA^ as a function of [KCTD11^BTB^]. (TIF)

S9 Fig The ^1^H NMR spectrum of Cul3 ^49-68SL^. (TIF)

S10 Fig Secondary Structure analysis. Summary of NMR parameters for Cul3^49-68EN^ (A) and Cul3^49-68LA^ (B). NOEs diagram, Chemical Shift Index (CSI) and Secondary Structure Propensities (SSP) are reported.
(TIF)

S11 Fig Cul3^49-68SL^. Overlay of a diagnostic portion of the NOESY spectrum of Cul3^49-68SL^ peptide (Blue) with the same region of Cul3^49-68EN^ spectrum (Red) showing in the case of Cul3^49-68SL^ peptide the absence of the typical α-helix NOE connectivities.
(TIF)

S1 Table ^1^H chemical shifts for Cul3^49-68^. (DOCX)

S2 Table ^1^H chemical shifts for Cul3^49-68LA^. (DOCX)

S3 Table ^1^H chemical shifts for Cul3^49-68EN^. (DOCX)

S4 Table Predictor results for Cul3^49-68EN^ peptide. (DOCX)

S5 Table Predictor results for Cul3^49-68EN^ peptide. (DOCX)
