# Supplementary material for: Cullin3 - BTB Interface: A Novel Target for Stapled Peptides
Source: PLoS One. 2015 Apr 7;10(4):e0121149. doi: 10.1371/journal.pone.0121149 (PMC4388676; doi:10.1371/journal.pone.0121149)
Supplement: S1 File — (DOCX) [file pone.0121149.s001.docx]

**Supporting information**

**Cullin3-BTB interface: a novel target for stapled peptides**

Ivan de Paola^1¶^, Luciano Pirone^2¶^, Maddalena Palmieri^3^, Nicole Balasco^1,3^, Luciana Esposito^1,4^, Luigi Russo^3^, Daniela Mazzà^5^, Lucia Di Marcotullio^5^, Sonia Di Gaetano^1,4^, Gaetano Malgieri^3^, Luigi Vitagliano^1,4^, Emilia Pedone^1,4,*^ and Laura Zaccaro^1,4*^

**S1 Information**

**Generation of the KCTD11^BTB^ three-dimensional model**

Two different crystallographic models were used as templates: (a) the structure of KCTD5^BTB^ pentamer which shares a high sequence identity with KCTD11^BTB^ (38%) but a different oligomeric state and (b) the structure of the BTB domain of akv3.1 voltage-gated potassium channel (PDB code 3KVT), which presents a lower sequence identity (33%) but the same tetrameric organization. In the initial stages of the modelling, KCTD11^BTB^ tetramer was generated using the tetrameric assembly of the akv3.1 BTB domain. We noticed, however, that the terminal helix of canonical one-sheet and five-helices motif of the BTB domains of akv3.1 does not share any sequence identity with the corresponding region of KCTD11^BTB^. Indeed, akv3.1 BTB is characterized, in this region, by the presence of residues that are involved in Zn coordination (Cys102 and Cys103). Since the metal binding motif is absent in KCTD11^BTB^, the helices α4 and α5 were modeled on KCTD5^BTB^.

**S2 Information**

**Protocol of the molecular dynamics simulation**

The model was immersed in a cubic box filled with 21861 water molecules. The dimensions of the box were 9.020x9.020x9.020 nm^3^. The OPLS all-atom force field and the TIP4P water model were used in the simulation. The simulation was run with periodic boundary conditions. Equilibration was conducted in two phases. The first phase was conducted under an NVT ensemble in order to stabilize the temperature of the systems at 300 K. Equilibration of pressure (1 atm) was then conducted under an NPT ensemble.

Before starting the MD simulation, energies were minimized by fixing the protein atoms and then without restraints. The system temperature was brought to 300 K in a step-wise procedure. In particular, 100 ps MD runs were carried out at 50, 100, 150, 200, 250, and 300 K. The timescale of the simulation was 130000 ps, with a time step of 0.002 ps. Bond lengths were constrained by the LINCS procedure. Lennard-Jones interactions were calculated with a 10 Å cutoff. Electrostatic interactions were treated using the Particle Mesh Ewald (PME) method (grid spacing of 0.12 nm). Trajectories were checked in order to assess the quality of the simulation using GROMACS routines and the program VMD.

**S3 Information**

**Monitoring of the structure stability of the models along the trajectory**

The evolution of the (KCTD11^BTB^-Cul3^49-68^)_4_ assembly during the simulation was monitored by using the indicators commonly adopted to check system stability in MD analyses. The trend of root mean square deviations (RMSD) of the trajectory structures from the starting model shows that the structure of complex undergoes to significant variations in the initial stages of the simulation (S2A Fig.). Trajectory structures reach, however, a rather stable state in the 30-130 ns interval (S2A Fig.). The average RMSD value, computed on the C^α^ atoms, in the plateau region is ~ 5.0 Å for KCTD11^BTB^. This value increases to ~ 6.0 Å for the overall complex. The analysis of the gyration radius of the complex indicates that (KCTD11^BTB^-Cul3^49-68^)_4_ undergoes to minor overall variations (S2B Fig.). The convergence of the simulation was evaluated through the analysis of the RMSIP value (see Methods for details). The 100 ns interval corresponding to the equilibrated trajectory (30-130 ns) was divided in two halves of 50 ns each. On both halves an essential dynamics analysis was performed. The RMSIP, computed considering the first ten eigenvectors derived from the diagonalization of the covariance matrices for each half, was 0.65. The good RMSIP value indicates that the essential subspace spanned by the first 10 eigenvectors of the two different sets considered is overlapped.
