# Supplementary figures and images for: Cullin3 - BTB Interface: A Novel Target for Stapled Peptides
Source: PLoS One. 2015 Apr 7;10(4):e0121149. doi: 10.1371/journal.pone.0121149 (PMC4388676; doi:10.1371/journal.pone.0121149)

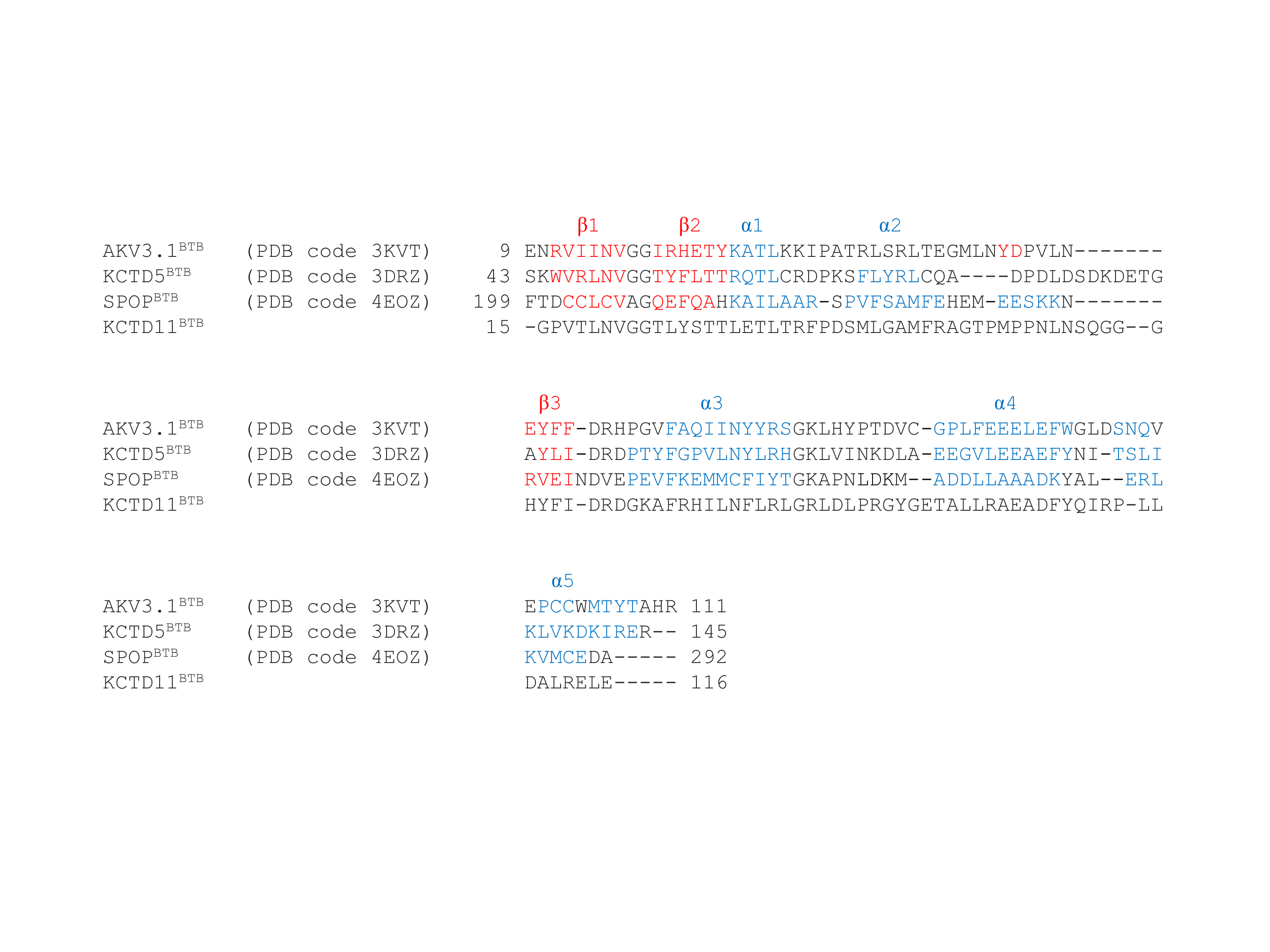

Supplement: S1 Fig — α-Helices and β-strands are denoted in blue and red, respectively. (TIF) [file pone.0121149.s002.tif]

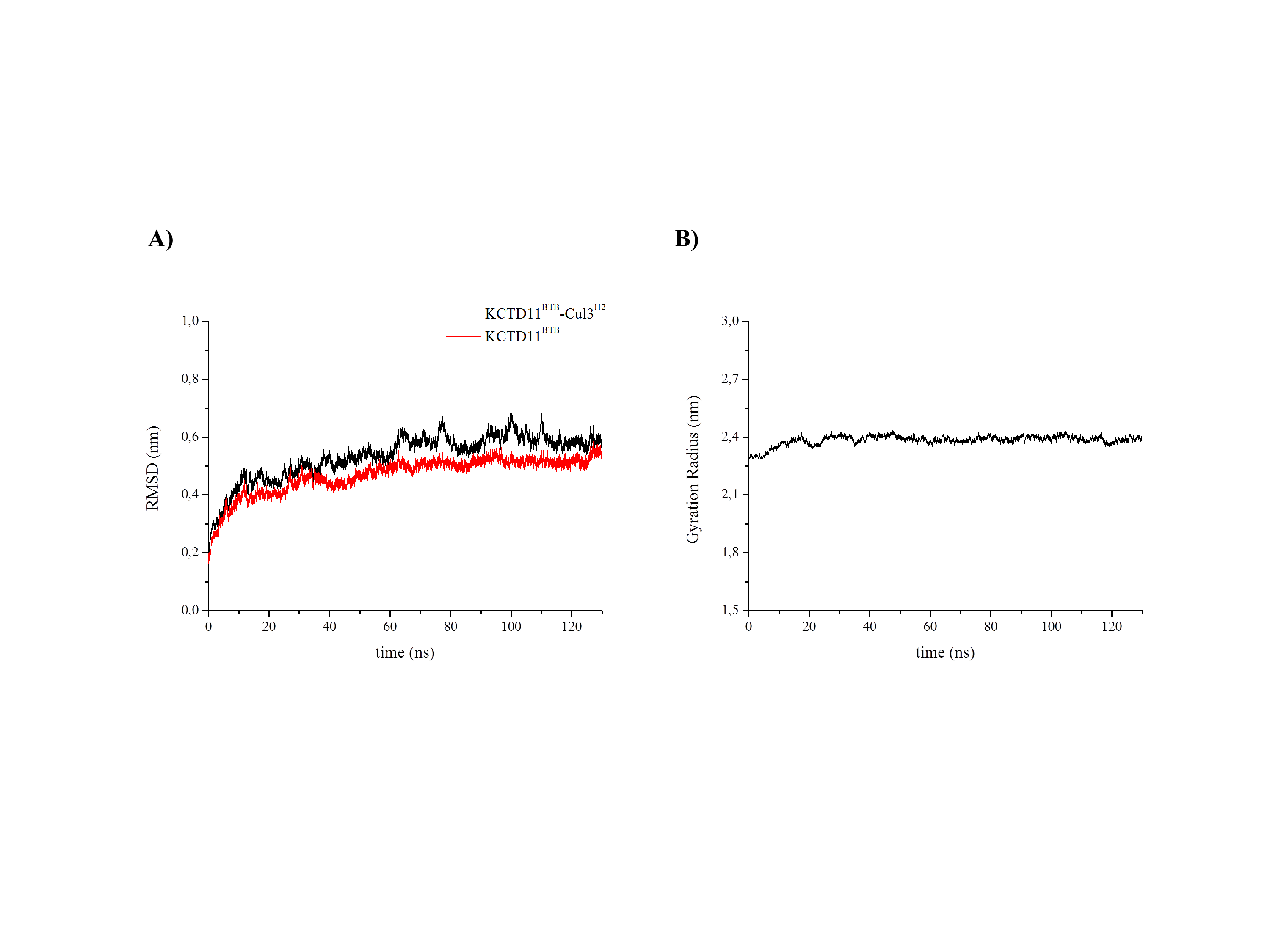

Supplement: S2 Fig — (A) Root mean square deviations, computed using Cα atoms, compared to the starting model and (B) gyration radius. (TIF) [file pone.0121149.s003.tif]

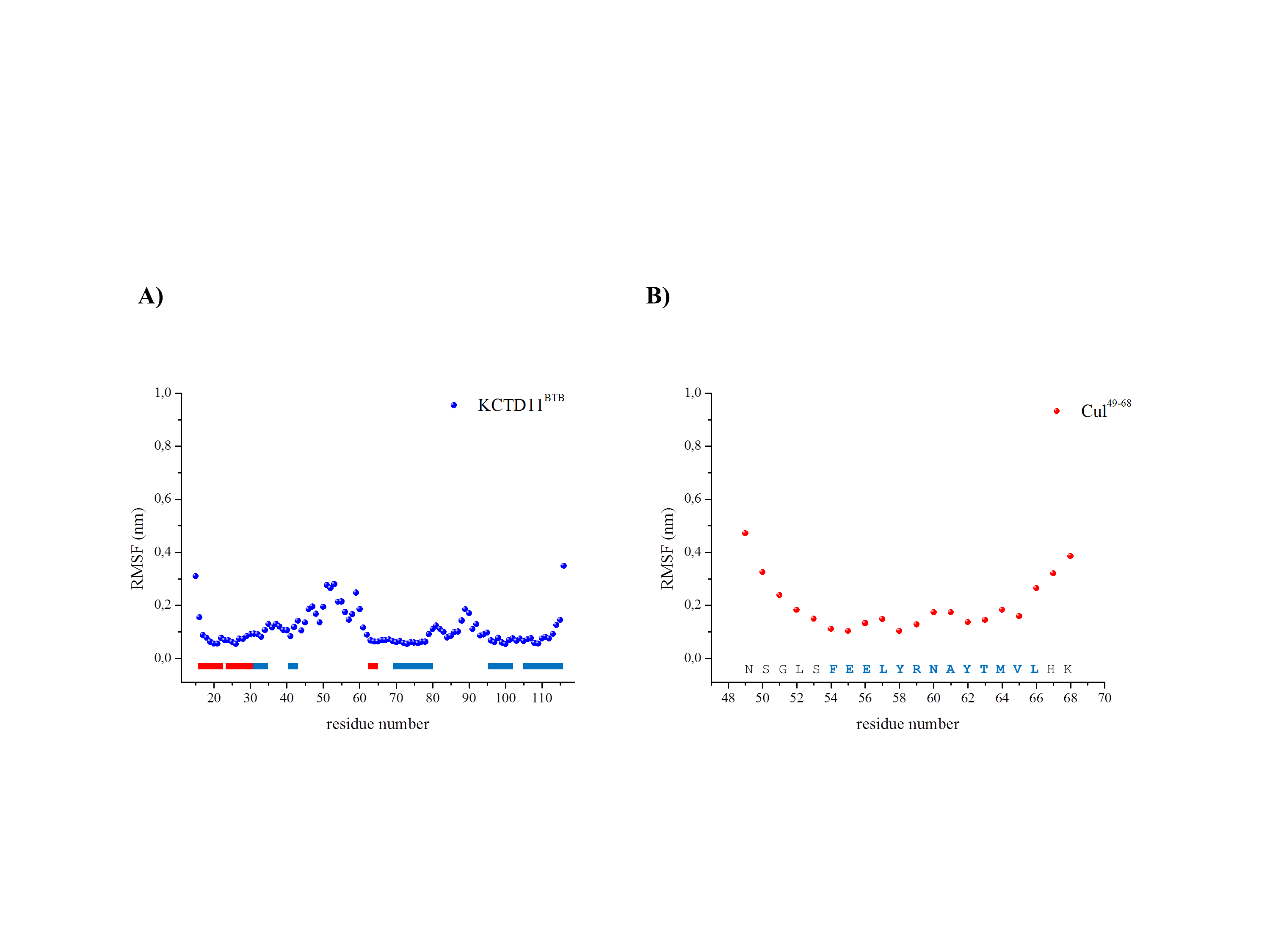

Supplement: S3 Fig — The lines reported in panels A represent residues in α-helices (cyan) or in β-sheets (red). In panel B, the helical residues of Cul349-68 are highlighted in cyan. (TIF) [file pone.0121149.s004.tif]

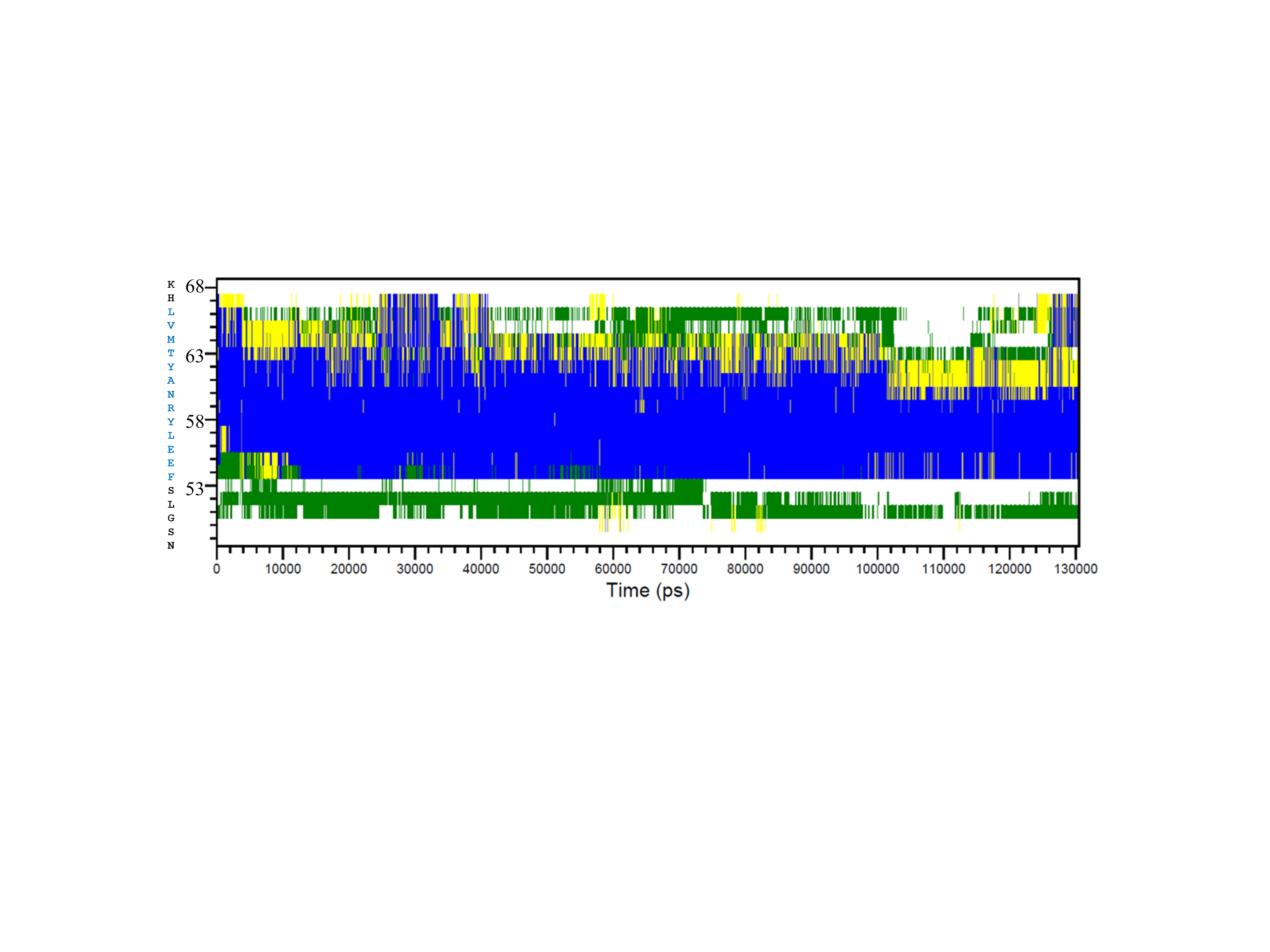

Supplement: S4 Fig — Regions in blue, grey, yellow and green represent α-helices, 3–10 helices, turns and bends, respectively. (TIF) [file pone.0121149.s005.tif]

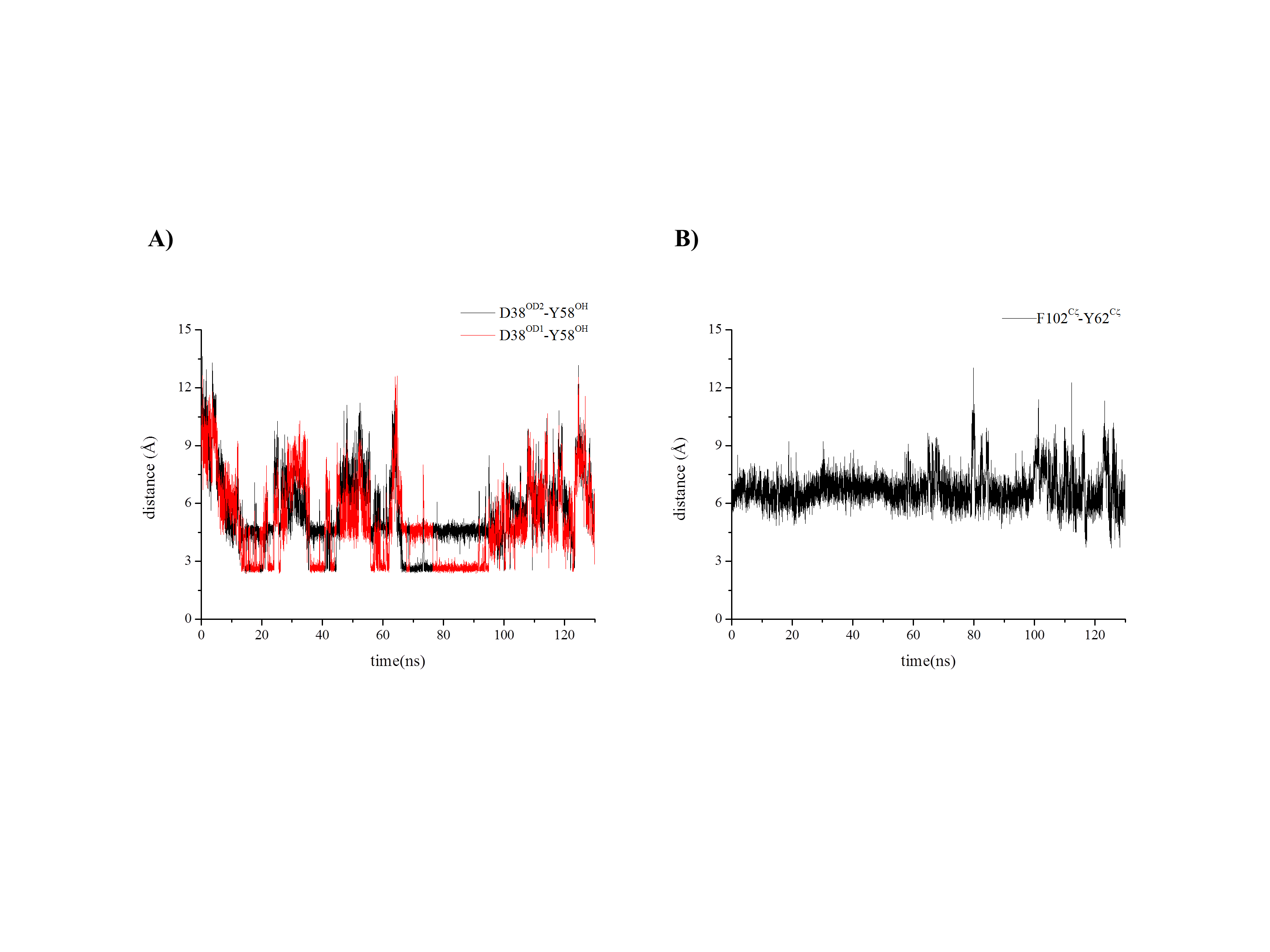

Supplement: S5 Fig — Examples of H-bonds and aromatic residues clustering are reported in panels A and B, respectively. (TIF) [file pone.0121149.s006.tif]

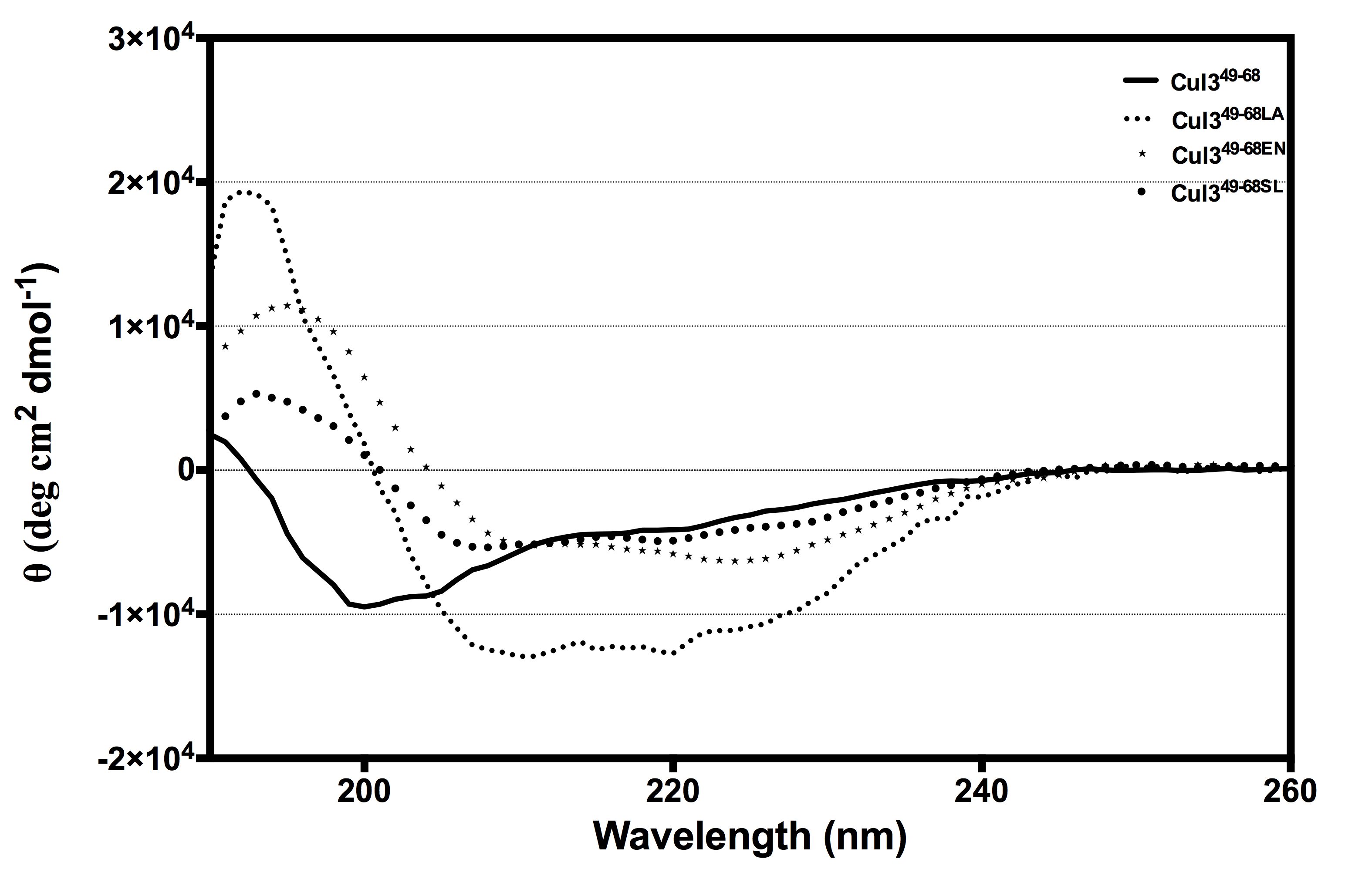

Supplement: S6 Fig — Spectra were acquired in 0,1% TFA (pH 3.0). (TIFF) [file pone.0121149.s007.tiff]

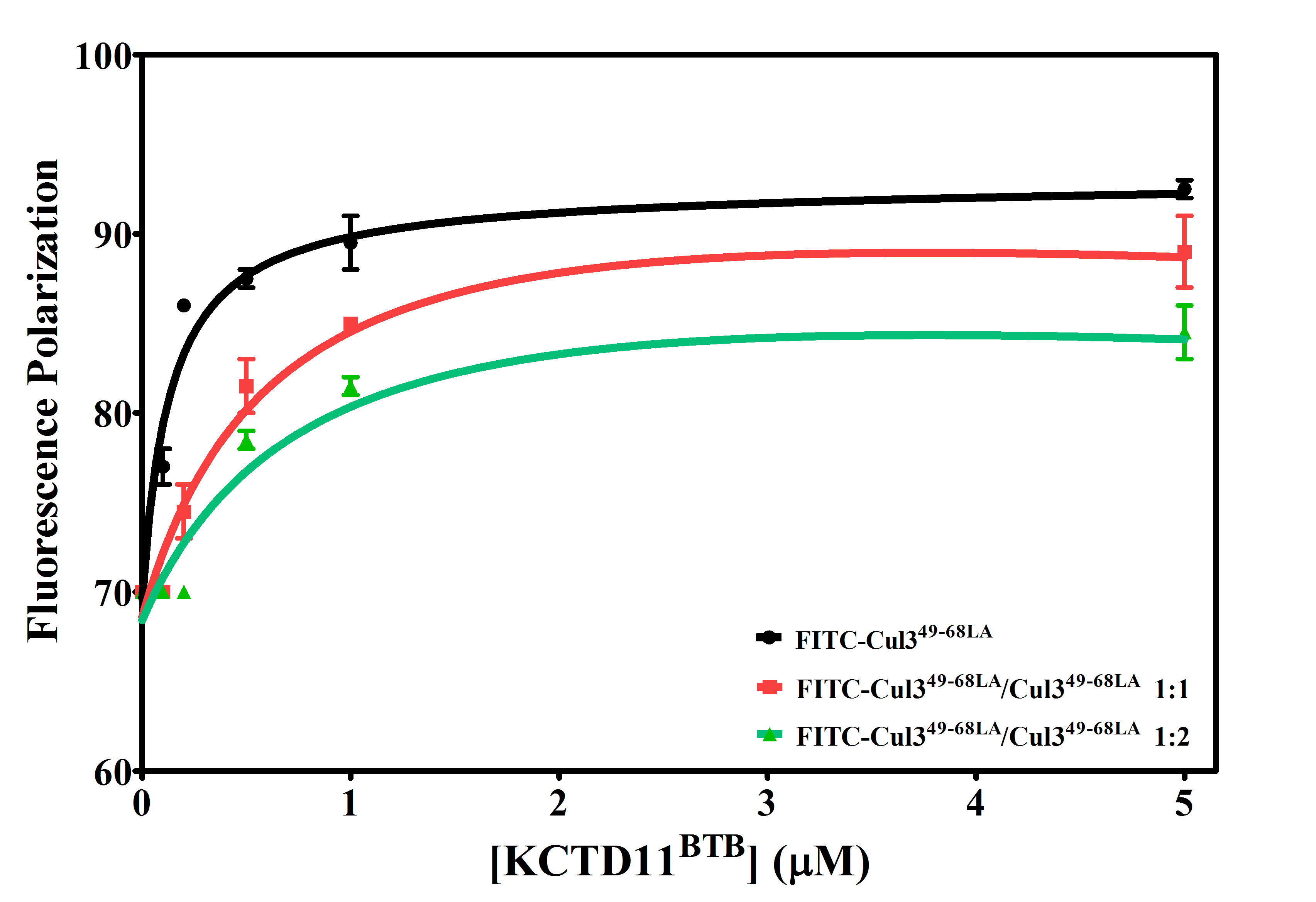

Supplement: S7 Fig — (TIF) [file pone.0121149.s008.tif]

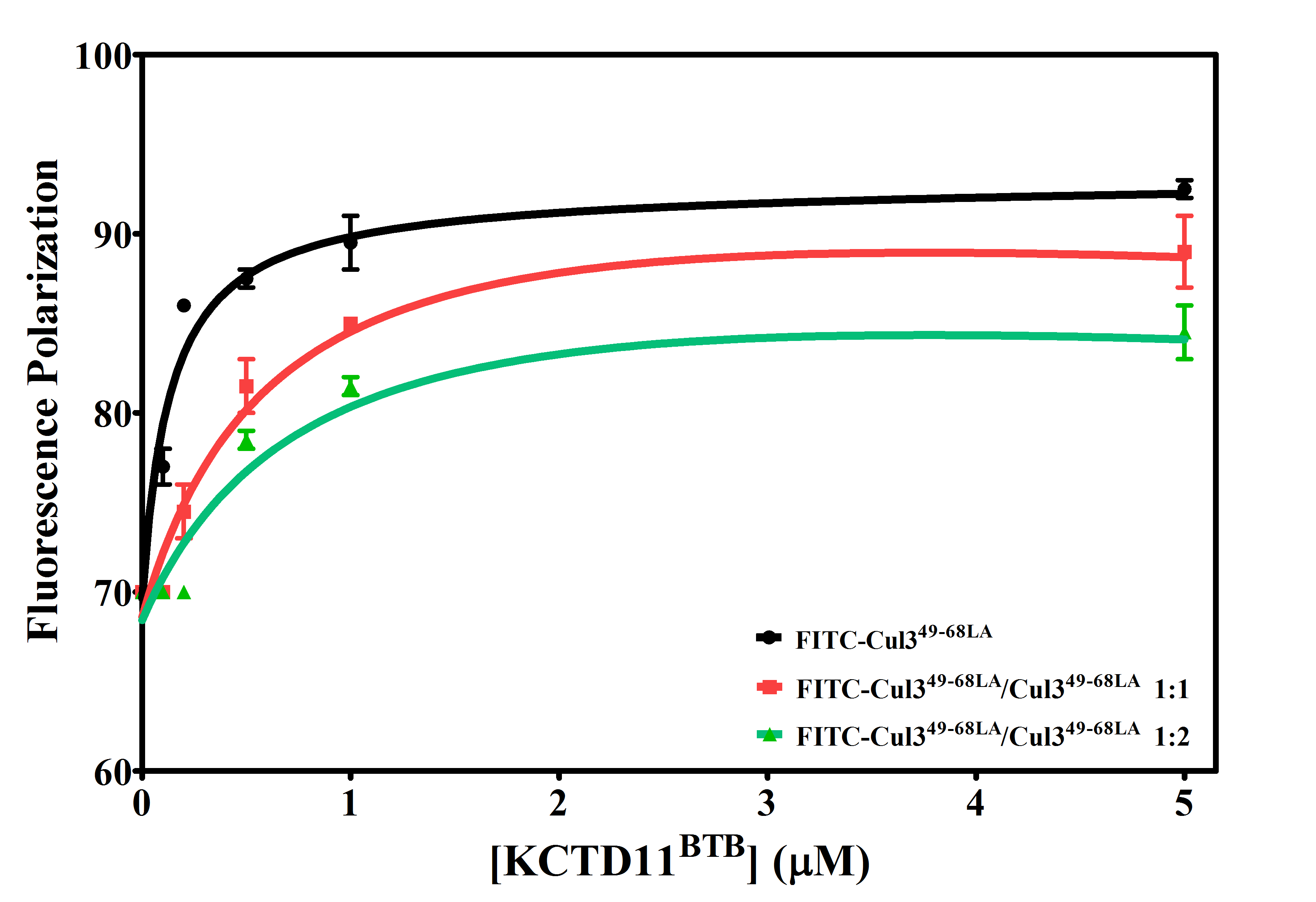

Supplement: S8 Fig — (TIF) [file pone.0121149.s009.tif]

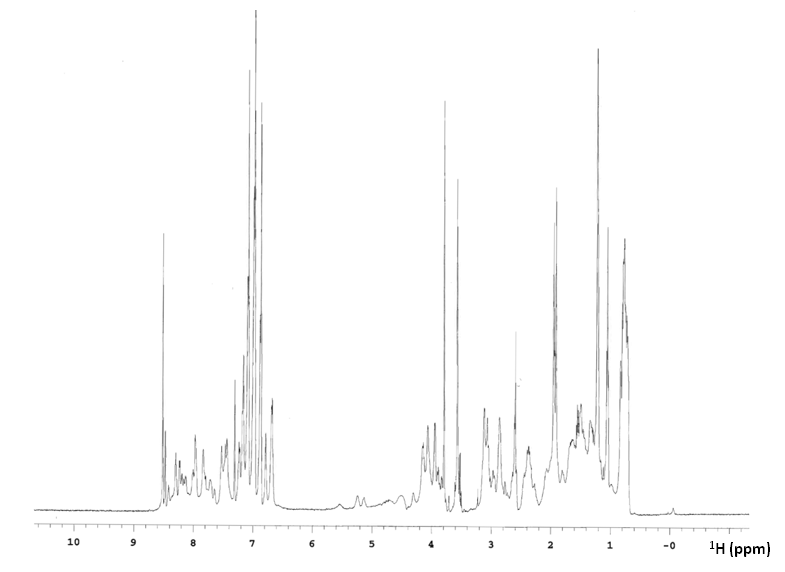

Supplement: S9 Fig — (TIF) [file pone.0121149.s010.TIF]

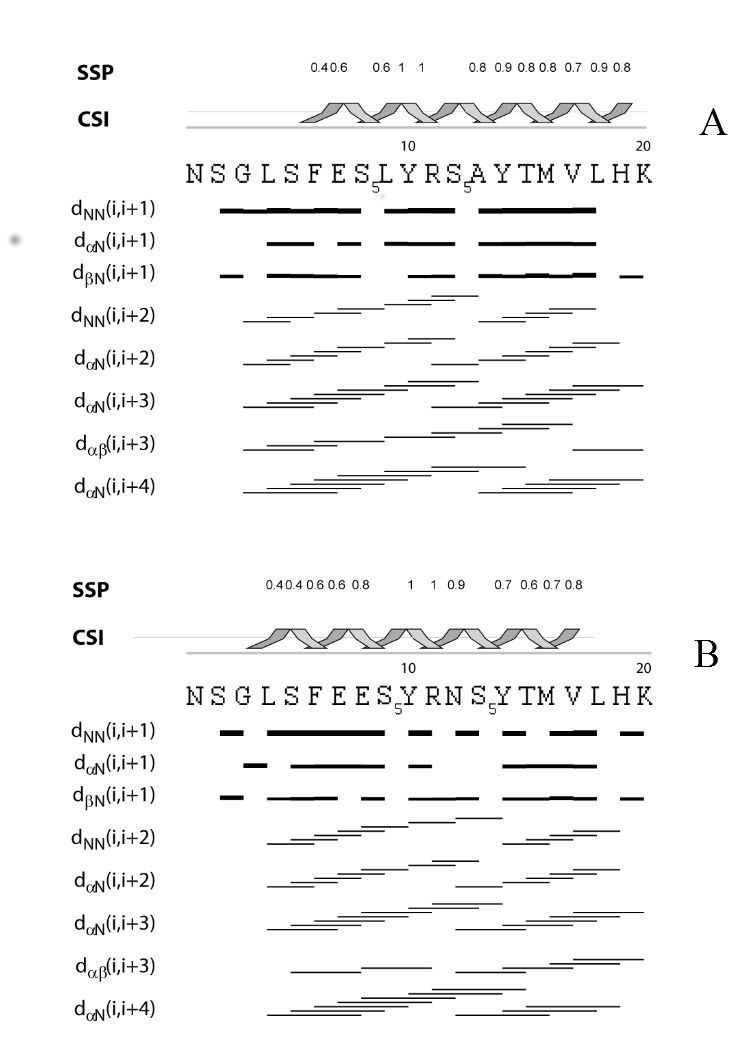

Supplement: S10 Fig — Summary of NMR parameters for Cul349-68EN (A) and Cul349-68LA (B). NOEs diagram, Chemical Shift Index (CSI) and Secondary Structure Propensities (SSP) are reported. (TIF) [file pone.0121149.s011.tif]

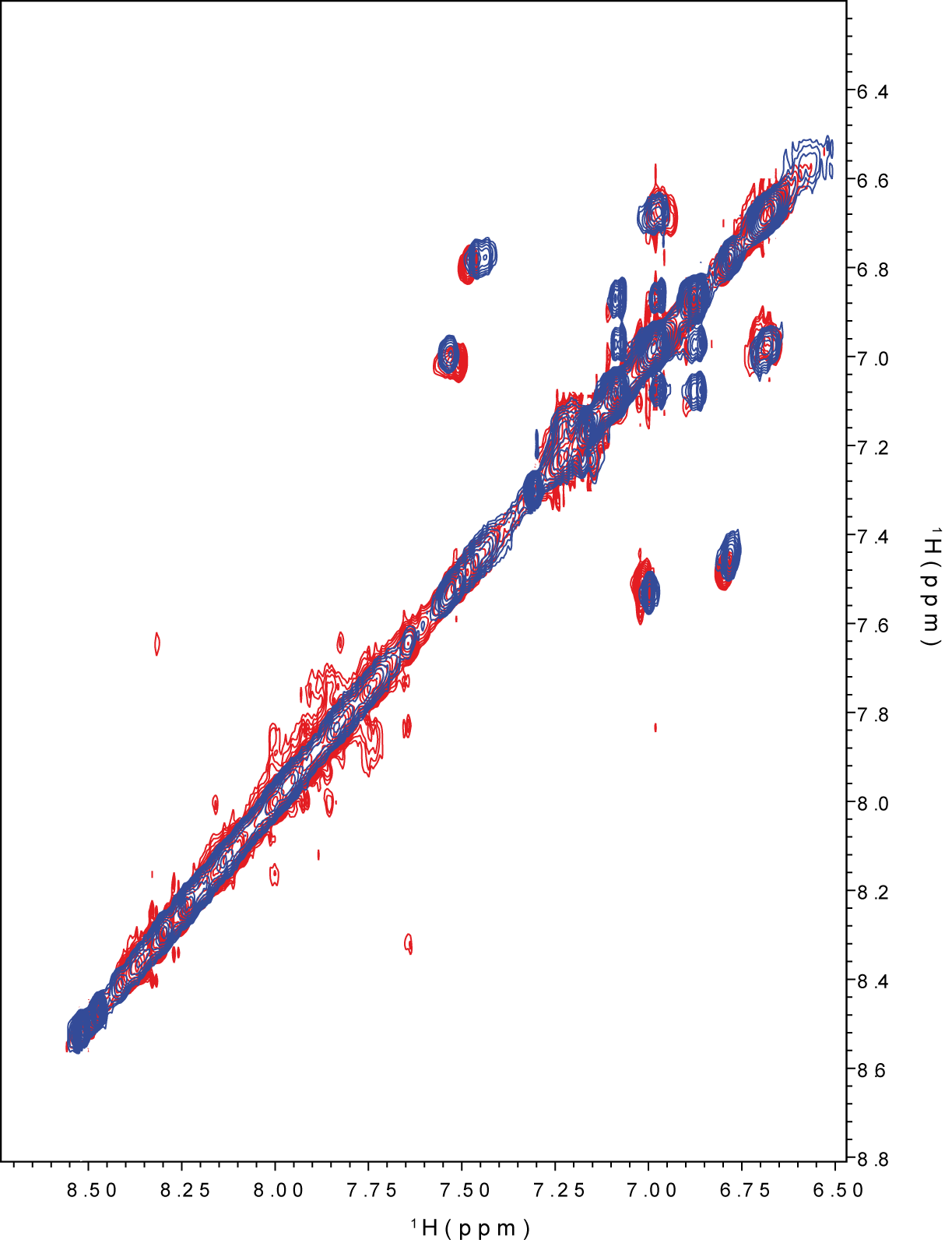

Supplement: S11 Fig — Overlay of a diagnostic portion of the NOESY spectrum of Cul349-68SL peptide (Blue) with the same region of Cul349-68EN spectrum (Red) showing in the case of Cul349-68SL peptide the absence of the typical α-helix NOE connectivities. (TIF) [file pone.0121149.s012.tif]
