# Supplementary material for: Cullin3 - BTB Interface: A Novel Target for Stapled Peptides
Source: PLoS One. 2015 Apr 7;10(4):e0121149. doi: 10.1371/journal.pone.0121149 (PMC4388676; doi:10.1371/journal.pone.0121149)
Supplement: S1 Table — (DOCX) [file pone.0121149.s013.docx]

| **RESIDUE** | **H_N_** | **H_α_** | **H_β_** | **H_γ_** | **H_δ_** | **H_ε_** |
| --- | --- | --- | --- | --- | --- | --- |
| ASN 1 | 8,25 | 4,56 | 2,63 |  |  |  |
| SER 2 | 8,11 | 4,24 | Hβ_2_ 3,79  Hβ_3_ 3,71 |  |  |  |
| GLY 3 | 8,24 | 4,11 |  |  |  |  |
| LEU 4 | 8,07 | 4,05 | 1,44 | 1,31 | 1,17 |  |
| SER 5 | 8,30 | 4,30 | 3,75 |  |  |  |
| PHE 6 | 8,34 | 3,84 | 1,91 |  | 7,88 | 7,48 |
| GLU 7 | 8,07 | 4,16 | 1,83 | 2,25 |  |  |
| GLU 8 | 8,04 | 4,29 | 1,89 | Hγ_2_ 2,39  Hγ_3_ 2,26 |  |  |
| LEU 9 | 7,93 | 3,88 | 1,37 | 1,22 | 0,78 |  |
| TYR 10 | 7,90 | 4,07 | Hβ_2_ 2,94  Hβ_3_ 2,78 |  | 6,93 | 6,64 |
| ARG 11 | 8,24 | 4,13 | Hβ_2_ 1,67  Hβ_3_ 1,58 | 1,28 | 2,85 | 7,42 |
| ASN 12 | 8,13 | 4,35 | Hβ_2_ 2,99  Hβ_3_ 2,64 |  |  |  |
| ALA 13 | 8,03 | 4,09 | 1,15 |  |  |  |
| TYR 14 | 7,99 | 4,43 | Hβ_2_ 2,94  Hβ_3_ 2,83 |  | 6,99 | 6,67 |
| THR 15 | 7,76 | 4,10 | 4,01 | 1,03 |  |  |
| MET 16 | 7,94 | 4,06 | 1,92 | 2,30 |  |  |
| VAL 17 | 7,88 | 3,90 | 1,88 | 0,73 |  |  |
| LEU 18 | 7,89 | 4,22 | 1,87 | 1,48 | 1,36 |  |
| HIS 19 | 8,35 | 4,54 | Hβ_2_ 3,12  Hβ_3_ 3,02 |  | Hδ_2_ 7,14 | Q_ε_ 8,47 |
| LYS 20 | 7,85 | 4,38 | 1,65 | 1,17 | 1,39 | Hε_2_ 2,91  Hε_3_ 2,71 |
