# Supplementary material for: Cullin3 - BTB Interface: A Novel Target for Stapled Peptides
Source: PLoS One. 2015 Apr 7;10(4):e0121149. doi: 10.1371/journal.pone.0121149 (PMC4388676; doi:10.1371/journal.pone.0121149)
Supplement: S2 Table — (DOCX) [file pone.0121149.s014.docx]

| **RESIDUE** | **H_N_** | **H_α_** | **H_β_** | **H_γ_** | **H_δ_** | **H_ε_** |
| --- | --- | --- | --- | --- | --- | --- |
| ASN 1 | 8,20 | 4,51 | 2,60 |  |  |  |
| SER 2 | 8,14 | 4,33 | Hβ_2_ 3,96  Hβ_3_ 3,79 |  |  |  |
| GLY 3 | 8,34 | 3,84 |  |  |  |  |
| LEU 4 | 7,88 | 4,24 | 1,68 | 1,61 | 0,89 |  |
| SER 5 | 8,30 | 4,28 | Hβ_2_ 3,78  Hβ_3_ 3,74 |  |  |  |
| PHE 6 | 8,25 | 4,59 | 2,64 |  | 7,68 | 7,28 |
| GLU 7 | 8,06 | 4,28 | 1,93 | 2,32 |  |  |
| GLU 8 | 7,82 | 3,80 | 1,96 | 2,36 |  |  |
| MK 9 | 7,73 | - | 1,89 | 1,26 | 2,30 |  |
| TYR 10 | 7,69 | 3,81 | Hβ_2_ 2,83  Hβ_3_ 2,60 |  | 6,91 | 6,69 |
| ARG 11 | 7,88 | 3,97 | 1,68 | 1,60 | 2,86 | 7,40 |
| ASN 12 | 8,06 | 3,78 | 2,97 |  |  |  |
| MK 13 | 8,14 | - | 1,98 | 1,43 | 2,31 |  |
| TYR 14 | 7,99 | 4,41 | Hβ_2_ 3,04  Hβ_3_ 2,85 |  | 6,94 | 6,64 |
| THR 15 | 7,87 | 4,07 | 3,96 | 1,46 |  |  |
| MET 16 | 7,93 | 4,22 | 2,00 | 2,40 |  |  |
| VAL 17 | 7,70 | 3,82 | 1,97 | Qγ_1_ 0,80  Qγ_2_ 0,72 |  |  |
| LEU 18 | 7,81 | 3,80 | 1,96 | 1,40 | 0,80 |  |
| HIS 19 | 8,00 | 4,53 | Hβ_2_ 3,02  Hβ_3_ 2,88 | - | Hδ_2_ 7,11 | 8,43 |
| LYS 20 | 7,06 | 4,07 | 1,81 | 1,51 | 1,65 | 3,06 |
