# Supplementary material for: Cullin3 - BTB Interface: A Novel Target for Stapled Peptides
Source: PLoS One. 2015 Apr 7;10(4):e0121149. doi: 10.1371/journal.pone.0121149 (PMC4388676; doi:10.1371/journal.pone.0121149)
Supplement: S3 Table — (DOCX) [file pone.0121149.s015.docx]

| **RESIDUE** | **H_N_** | **H_α_** | **H_β_** | **H_γ_** | **H_δ_** | **H_ε_** |
| --- | --- | --- | --- | --- | --- | --- |
| ASN 1 | 7,73 | 4,01 | Hβ_2_ 2,90  Hβ_3_ 2,78 |  |  |  |
| SER 2 | 7,92 | 4,20 | Hβ_2_ 3,74  Hβ_3_ 3,66 |  |  |  |
| GLY 3 | 8,19 | 4,11 |  |  |  |  |
| LEU 4 | 7,88 | 4,21 | 1,44 | 1,30 | 0,72 |  |
| SER 5 | 8,30 | 4,30 | 3,76 |  |  |  |
| PHE 6 | 8,36 | 3,85 | 1,89 |  | 7,87 | 7,45 |
| GLU 7 | 8,24 | 4,57 | 2,61 | 2,12 |  |  |
| MK 8 | 7,82 | - | 1,84 | 1,51  1,08 | 2,32 |  |
| LEU 9 | 7,91 | 4,08 | 1,47 | 1,35 | 0,70 |  |
| TYR 10 | 7,90 | 4,08 | Hβ_2_ 2,94  Hβ_3_ 2,68 |  | 6,92 | 6,60 |
| ARG 11 | 8,09 | 4,12 | Hβ_2_ 1,69  Hβ_3_ 1,58 | 1,28 | 2,84 | 7,42 |
| MK 12 | 7,76 | - | 1,09 |  | 1,90 |  |
| ALA 13 | 7,73 | 3,85 | 1,10 |  |  |  |
| TYR 14 | 8,00 | 4,27 | Hβ_2_ 2,94  Hβ_3_ 2,79 |  | 6,92 | 6,63 |
| THR 15 | 7,84 | 4,21 | 3,89 | 1,18 |  |  |
| MET 16 | 7,98 | 3,86 | 1,84 | 2,29 |  |  |
| VAL 17 | 7,75 | 3,86 | 1,93 | Qγ_1_ 0,77  Qγ_2_ 0,70 |  |  |
| LEU 18 | 7,74 | 4,02 | 1,11 | 1,09 | 0,68 |  |
| HIS 19 | 8,11 | 4,52 | Hβ_2_ 3,13  Hβ_3_ 3,04 | - | Hδ_2_ 7,14 | 8,45 |
| LYS 20 | 7,07 | 3,86 | Hβ_2_ 1,70  Hβ_3_ 1,80 | 1,47 | 1,58 | 3,04 |
