# Supplementary material for: Cullin3 - BTB Interface: A Novel Target for Stapled Peptides
Source: PLoS One. 2015 Apr 7;10(4):e0121149. doi: 10.1371/journal.pone.0121149 (PMC4388676; doi:10.1371/journal.pone.0121149)
Supplement: S4 Table — (DOCX) [file pone.0121149.s016.docx]

| **AA** | **ϕ** | **Error** | **ψ** | **Error** |
| --- | --- | --- | --- | --- |
| N | ------- | ---------------- |  | ---------------- |
| S | -54.2 | 85.4 | -25.6 | 116.0 |
| G | 117.7 | 85.4 | -34.2 | 116.0 |
| L | -65.3 | 71.2 | -38.1 | 97.3 |
| S | -64.6 | 71.2 | -37.7 | 97.3 |
| F | -67.7 | 56.9 | -42.6 | 78.6 |
| E | -102.7 | 56.9 | 79.1 | 78.6 |
| S_5_ | ------- | --------------- | ------- | --------------- |
| L | -61.0 | 56.9 | -42.9 | 78.6 |
| Y | -64.5 | 56.9 | -44.2 | 78.6 |
| R | -62.3 | 71.2 | -42.4 | 97.3 |
| S_5_ | ------- | --------------- | ------- | --------------- |
| A | -66.5 | 71.2 | -36.9 | 97.3 |
| Y | -62.4 | 71.2 | -42.1 | 97.3 |
| T | -64.4 | 71.2 | -43.8 | 97.3 |
| M | -67.2 | 56.9 | -43.5 | 78.6 |
| V | -60.4 | 71.2 | -43.5 | 97.3 |
| L | -59.4 | 71.2 | -42.7 | 97.3 |
| H | -62.4 | 71.2 | -40.9 | 97.3 |
| K | ------- | --------------- | ------- | --------------- |
