# Supplementary material for: Cullin3 - BTB Interface: A Novel Target for Stapled Peptides
Source: PLoS One. 2015 Apr 7;10(4):e0121149. doi: 10.1371/journal.pone.0121149 (PMC4388676; doi:10.1371/journal.pone.0121149)
Supplement: S5 Table — (DOCX) [file pone.0121149.s017.docx]

| **AA** | **ϕ** | **Error** | **ψ** | **Error** |
| --- | --- | --- | --- | --- |
| N | ------- | ---------------- | ------- | ---------------- |
| S | -68.5 | 85.4 | -39.3 | 116.0 |
| G | -66.4 | 85.4 | -41.2 | 116.0 |
| L | -63.2 | 85.4 | -40.9 | 116.0 |
| S | -67.2 | 85.4 | -39.8 | 116.0 |
| F | -65.0 | 85.4 | -38.2 | 116.0 |
| E | -68.1 | 85.4 | 144.8 | 116.0 |
| E | -62.2 | 71.2 | -34.1 | 97.3 |
| S_5_ | ------- | ---------------- | ------- |  |
| Y | -62.4 | 56.9 | -46.0 | 78.6 |
| R | -66.0 | 56.9 | -40.4 | 78.6 |
| N | -66.4 | 56.9 | -38.0 | 78.6 |
| S_5_ | ------- | ---------------- | ------- | ---------------- |
| Y | -67.7 | 71.2 | -37.7 | 97.3 |
| T | -61.9 | 71.2 | -43.0 | 97.3 |
| M | -64.8 | 71.2 | -40.9 | 97.3 |
| V | -65.3 | 71.2 | -41.9 | 97.3 |
| L | -62.6 | 71.2 | -38.9 | 97.3 |
| H | -66.4 | 71.2 | -35.5 | 97.3 |
| K | ------- | ---------------- | ------- | ---------------- |
